# Selective Inhibition of the Mitochondrial Permeability Transition Pore Protects against Neurodegeneration in Experimental Multiple Sclerosis*[Fn FN2]

**DOI:** 10.1074/jbc.M115.700385

**Published:** 2015-12-17

**Authors:** Justin Warne, Gareth Pryce, Julia M. Hill, Xiao Shi, Felicia Lennerås, Fabiola Puentes, Maarten Kip, Laura Hilditch, Paul Walker, Michela I. Simone, A. W. Edith Chan, Greg J. Towers, Alun R. Coker, Michael R. Duchen, Gyorgy Szabadkai, David Baker, David L. Selwood

**Affiliations:** From the ‡ Wolfson Institute for Biomedical Research, University College London, Gower Street, London WC1E 6BT, United Kingdom,; the § Neuroimmunology Unit, Blizard Institute, Barts and the London School of Medicine and Dentistry, Queen Mary University of London, 4 Newark Street, London E1 2AT, United Kingdom,; the ¶ Department of Cell and Developmental Biology, University College London, London WC1E 6BT, United Kingdom,; the ‖ Medical Research Council Centre for Medical Molecular Biology, Division of Infection and Immunity, University College London, London WC1E 6BT, United Kingdom,; ** Cyprotex Discovery Ltd., 100 Barbirolli Square, Manchester M2 3AB, United Kingdom, and; the §§ Department of Biomedical Sciences, University of Padua, Padua 35122, Italy

**Keywords:** cyclophilin D, mitochondrial permeability transition (MPT), multiple sclerosis, neurodegeneration, neurodegenerative disease, X-ray crystallography, EAE, cyclosporin, mitochondrial targeting

## Abstract

The mitochondrial permeability transition pore is a recognized drug target for neurodegenerative conditions such as multiple sclerosis and for ischemia-reperfusion injury in the brain and heart. The peptidylprolyl isomerase, cyclophilin D (CypD, PPIF), is a positive regulator of the pore, and genetic down-regulation or knock-out improves outcomes in disease models. Current inhibitors of peptidylprolyl isomerases show no selectivity between the tightly conserved cyclophilin paralogs and exhibit significant off-target effects, immunosuppression, and toxicity. We therefore designed and synthesized a new mitochondrially targeted CypD inhibitor, JW47, using a quinolinium cation tethered to cyclosporine. X-ray analysis was used to validate the design concept, and biological evaluation revealed selective cellular inhibition of CypD and the permeability transition pore with reduced cellular toxicity compared with cyclosporine. In an experimental autoimmune encephalomyelitis disease model of neurodegeneration in multiple sclerosis, JW47 demonstrated significant protection of axons and improved motor assessments with minimal immunosuppression. These findings suggest that selective CypD inhibition may represent a viable therapeutic strategy for MS and identify quinolinium as a mitochondrial targeting group for *in vivo* use.

## Introduction

A considerable body of evidence points to a role for the mitochondrial permeability transition (PT)^7^ pore in neurodegenerative and ischemic cell death. The peptidylprolyl *cis-trans*-isomerase cyclophilin D (CypD, PPIF), which is genomically expressed and imported into mitochondria, is consistently implicated as a key player in the sequence of events leading to PT pore opening and eventual cell death by necrosis. The PT pore forms under conditions of oxidative stress, low adenine nucleotide concentrations, and mitochondrial Ca^2+^ overload and results in free passage of low molecular mass solutes (<1500 Da) and some proteins across the inner mitochondrial membrane. Under these conditions, mitochondrial proton gradient and membrane potential (ψ_m_) are dissipated, leading to ATP hydrolysis by the reversal of the F_1_F_0_-ATP synthase and consequent cellular energy depletion, resulting in cell death. Recent studies point to the F_1_F_0_-ATP synthase of mitochondria as being the major component of the PT pore (1), but the subunits involved and the exact pore forming mechanism are controversial (2–4). CypD binds to the lateral stalk of the F_1_F_0_-ATPase and positively regulates pore opening (5, 6). In CypD knock-out animals, the pore is desensitized to Ca^2+^, in an inorganic phosphate (P_i_)-dependent manner (7). There is mounting evidence that CypD is key in mediating Ca^2+^-induced pore opening, and its absence (*e.g.* in PPIF knock-out animals) desensitizes the pore to Ca^2+^, in an inorganic phosphate (P_i_)-dependent manner (7). Pharmacological inhibition of the pore offers a route to cyto- and neuroprotection.

Multiple sclerosis (MS) is an immunomediated demyelinating and neurodegenerative disease of the central nervous system and the commonest form of non-traumatic disability in young adults (8). Although relapsing autoimmunity in MS can be controlled by peripheral immunomodulatory agents, progressive disability that results from neurodegeneration is, so far, untreatable (8, 9). Neurodegeneration in MS is associated with the influence of centrally active inflammatory responses (10, 11). This may relate to metabolic and energy stresses in nerves within the inflammatory penumbra that drive nerve loss during neuroinflammation in MS and other neurodegenerative diseases (12–14). Mitochondrial dysfunction and the irreversible opening of the PT pore are now recognized as a key players in the degeneration of axons (15). In MS lesions (12, 16, 17), the PT pore-induced ATP deficit may result in the inactivationof energy-dependent sodium/potassium pumps, leading to sodium loading and the reversal of the sodium-calcium exchanger that causes toxic accumulation of calcium ions and the induction of cell death effector pathways (16, 18).

CypD is highly expressed in a subset of astrocytes, microglia, and neurons (19), where it may contribute to excitotoxicity and cell death in MS lesions (12, 16, 17). CypD knock-out mice show a less severe phenotype compared with wild type in the experimental autoimmune encephalomyelitis (EAE) model of MS (20, 21). CypD knock-out mouse studies in models of traumatic brain injury (22, 23), Alzheimer disease (24, 25), Parkinson disease (26), amyloid lateral sclerosis (27), and Huntington disease (28, 29), all show a benefit compared with wild type mice. The PT pore is also implicated in ischemia-reperfusion injury in the adult brain (30) and in the heart, where CypD ablation or RNAi knockdown (31, 32) provides cardio-protection (33, 34). A selective inhibitor of PT pore opening could therefore have therapeutic applicability in a range of diseases, particularly MS, where the progressive disability that results from neurodegeneration is so far untreatable (8, 9).

**FIGURE 1.**
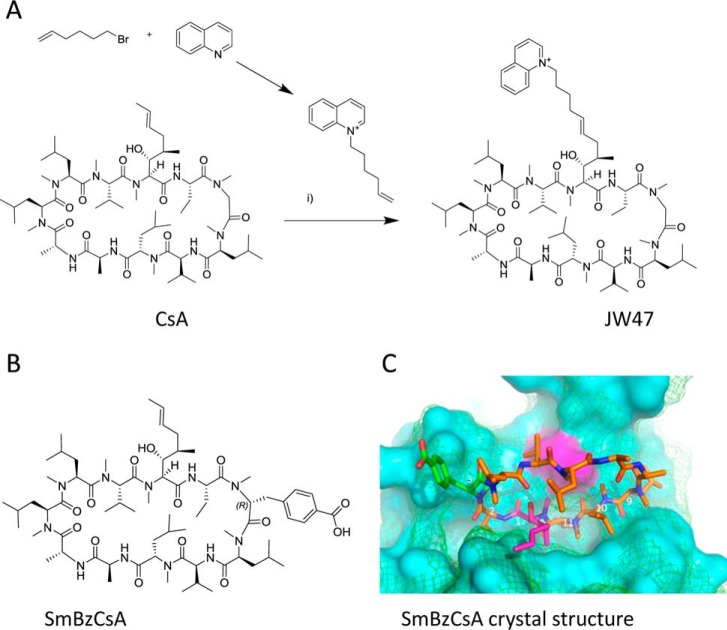
*A*, synthesis of [Bmt^1^]- and [Sar^3^]-substituted analogues by olefin cross-metathesis and alkylation. Reagents were DCM, Hoveyda-Grubbs catalyst, second generation, reflux, 30 h (*i*) and ethyl acetate reflux (*ii*). *B*, structure of SmBzCsA. *C*, the crystal structure of SmBzCsA (Protein Data Bank code 4IPZ) is shown bound to CypA (*solid magenta surface*). The aligned structure of CypD (Protein Data Bank code 5A0E) is shown as a *wire mesh*. Amino acids 9 to 2 are involved in cyclophilin binding. The [Bmt^1^] residue (*pink*) side chain and the [Sar^3^] substituent do not affect cyclophilin binding.

Cyclosporine (cyclosporin A (CsA); Fig. 1*A*) is a non-selective cyclophilin inhibitor. CsA forms a ternary complex with the cytoplasmic CypA and calcineurin, leading to inhibition of calcineurin signaling. This blocks downstream cytokine production associated with immune cell activation (35, 36) and makes CsA a potent and clinically useful immunosuppressive. Chemical modification to remove calcineurin binding is relatively straightforward, but selectivity for the different cyclophilin proteins is difficult due to their close structural and sequence similarity (37). Neuroprotective actions of CsA via action on mitochondrial CypD are reported; case studies and clinical trials support a neuroprotective effect (38, 39), whereas clinical studies with CsA in traumatic brain injury are in progress (NCT01825044). *In vitro* CsA shows cytotoxicity and multiple effects on cell health parameters, whereas problems with the clinical use of CsA are nephrotoxicity (35, 39), bilirubinemia, and liver toxicity (40), which can require withdrawal of the drug. These properties combine to make CsA a less than ideal drug candidate for neuroprotection.

A potential solution to the problem of targeting the individual cyclophilins is to enable mitochondrial localization. Triphenylphosphonium (TPP^+^) is the archetypal mitochondrial targeting, lipophilic cation (41) and has been used in humans for targeting a coenzyme Q analogue to mitochondria (42). TPP^+^ has non-ideal pharmaceutical properties however, including (*a*) high molecular weight and lipophilicity, contributing to a lack of “drug likeness”; (*b*) mitochondrial toxicity (43, 44); (*c*) effects on respiration (45); and (*d*) non-ideal biodistribution (46). We have previously shown that TPP^+^ can be linked to CsA at the [Sar]^3^ position to provide a molecule with immunosuppression blocked and improved cytoprotection *in vitro* (36, 47).

Here we investigated the quinolinium cation as a replacement for triphenylphosphonium. We observed that quinolinium is an effective mitochondrial targeting group; a prototype molecule, JW47, was shown to be more potent at blocking the PT pore and demonstrated less cell toxicity than CsA. *In vivo* JW47 was less immunosuppressive than CsA and notably achieved significant neuroprotection in an EAE model of MS in mice.

## Experimental Procedures

### 

#### 

##### Chemistry

All commercially available solvents and reagents were used without further treatment as received unless otherwise noted. NMR spectra were measured with a Bruker DRX 500- or 600-MHz spectrometer; chemical shifts are expressed in ppm relative to TMS as an internal standard, and coupling constants (*J*) are reported in Hz. LC-MS spectra were obtained using a Waters ZQ2000 single quadrupole mass spectrometer with electrospray ionization, using an analytical C4 column (Phenomenex Gemini; 50 × 3.6 mm, 5 μm) and an AB gradient of 50–95% B at a flow rate of 1 ml/min, where eluent A was 0.1:5:95 formic acid/methanol/water, and eluent B was 0.1:5:95 formic acid/water/methanol. High resolution mass spectra were acquired on a Waters LCT time-of-flight mass spectrometer with electrospray ionization or chemical ionization.

##### Preparation of JW47, 1-(Pent-4-enyl)quinolinium

To a solution of quinoline (1 g, 7.74 mmol) in EtOAc was added 5-bromo-pent-1-ene (1.27 g, 8.51 mmol), and this mixture was heated to reflux overnight. The mixture was allowed to cool before concentration under reduced pressure. The product was isolated as a light brown oil (1.54 g, 99%).

LC-MS (*m*/*z*): [MH]^+^ calcd. for C_14_H_16_N+, 198.29; found 198.10. NMR δ_H_ (acetone-*d*_6_, 600 MHz): δ 10.26 (dd, *J* = 5.8, 1.4 Hz, 1H), 9.41 (d, *J* = 8.4 Hz, 1H), 8.80 (d, *J* = 9.0 Hz, 1H), 8.58 (dd, *J* = 8.2, 1.3 Hz, 1H), 8.36 (dd, *J* = 8.3, 1.5 Hz, 1H), 8.27 (dd, *J* = 8.3, 5.8 Hz, 1H), 8.13–8.08 (m, 1H), 5.90 (dd, *J* = 17.0, 10.3 Hz, 1H), 5.49–5.42 (m, 2H), 5.09 (ddd, *J* = 17.1, 3.4, 1.6 Hz, 1H), 1H), 5.01–4.96 (m, 1H), 2.41–2.35 (m, 2H), 2.34–2.26 (m, 2H).

##### [Gly-(1S,2R,E)-8-quinolinium-1-hydroxy-2-methyloct-4-ene]^1^ CsA (JW47)

To a solution of cyclosporin A (75 mg, 0.06 mmol) in DCM (2 ml) was added 1-(pent-4-en-1-yl)quinolinium (23 mg, 0.072 mmol) and Hoveyda-Grubbs second generation catalyst (7 mg, 0.01 mmol, 17 mol %). The reaction was stirred in the microwave at 90 °C for 30 min and then allowed to cool. Triethylamine was added to the mixture and then stirred overnight with excess P(CH_2_OH)_3_ to coordinate the ruthenium catalyst. This was then washed away with brine and water before the mixture was passed through a Stratospheres PL Thiol MP SPE cartridge (polymer laboratory, Varian Inc.) to remove any remaining catalyst. The crude product was purified by flash reverse-phase chromatography (MeOH/H_2_O/formic acid) to give JW47 as a brown solid (15 mg, 17%). HRMS (*m/z*): [MH]^+^ calcd. for C_65_H_115_N_11_O_12_, 1357.92; found 1357.95. NMR δ_H_ (CDCl_3_, 600 MHz): δ 3.49 (s, NMe, 3H), 3.40 (s, NMe, 3H), 3.20 (s, NMe, 3H), 3.12 (s, NMe, 3H), 3.08 (s, NMe, 3H), 2.71 (s, NMe, 3H), 2.68 (s, NMe, 3H).

NMR δ_C_ (CDCl_3_, 150 MHz): 39.35, 39.19, 33.67, 31.25, 30.10, 30.01, 29.79 (7 × N-Me).

##### Protein Expression

The CypD expression system was constructed as described by Schlatter *et al.* (48). Briefly, codon-optimized DNA encoding the CypD-K133I gene was cloned into pET11a (Novogen) digested with NdeI and BamHI. The resulting plasmid was used to transform BL21(DE3)pLysS. Expression cultures were grown at 37 °C in Luria-Bertani (LB) medium, at *A*_600_ of 0.6, and the cells were induced with 1 mm isopropyl-d-1-thiogalactopyranoside. The cultures were then incubated at the same temperature for another 4 h, and the cells were harvested by centrifugation. The cell pellet was resuspended in lysis buffer (100 mm Tris/HCl, pH 7.8, 2 mm EDTA, 2 mm DTT supplemented with EDTA-free protease inhibitor tablets (Roche Applied Science)), and the cells were lysed by sonication. The sonicate was clarified by centrifugation, and the CypD was isolated as described previously (49) using ion exchange chromatography with SP-Sepharose followed by Q-Sepharose and finally size exclusion chromatography with Superdex200. The protein was stored in size exclusion chromatography buffer (50 mm potassium/sodium phosphate, pH 7.3, 100 mm NaCl, 2 mm EDTA, 0.02% sodium azide) at 4 °C.

##### Crystallography

Crystals were grown using the hanging drop method as described previously (50); drops contained a 50:50 mix of protein solution (30 mg/ml CypD in gel filtration buffer) and reservoir solution. The best crystals were obtained with a reservoir solution consisting of 23% polyethylene glycol (PEG 3350), 50 mm sodium citrate buffer at pH 2.9 as precipitant solution. Prior to data collection, the crystals were cooled to 100 K using 35% PEG 3350 in sodium citrate buffer at pH 2.9 as the cryoprotectant. Data were collected on beamline i04 at Diamond Light Source. Phases were determined by the molecular replacement method using PHASER (51) from the CCP4 suite of programs (52); the CypD-cyclosporin A structure (Protein Data Bank code 2Z6W) was used as the search model but with the cyclosporin A removed from the model. The model was refined using cycles of the program REFMAC (53, 54) (with anisotropic *B*-factors and Babinet bulk solvent modeling), interspersed with manual checks and model building with COOT (55). The final resolution cut-off was determined during refinement. Ligand topologies were generated with PRODRG2 (56). The final model was validated with PROCHECK (57) and SFCHECK (54) and finally MOLPROBITY (58). Refinement statistics are presented in Table 1. The illustrations were produced with the PyMOL molecular graphics system (Shrödinger LLC, New York).

##### Fluorescence Polarization Assay

Fluorescence polarization (FP) is inversely related to the molecular rotation of the fluorophore. Fluorophores linked to small molecules tumble faster and emit less polarized fluorescence than fluorophore-small molecules bound to proteins. FP is a convenient technique to measure affinity of ligands to cyclophilins (59, 60). We synthesized a new fluorescein-PEG-CsA ligand as the probe (see above), and we determined the binding of our novel ligands to CypD and CypA.

Titration of a single probe concentration against different enzyme concentrations was used to determine the dissociation constant (*K_d_*). From this we also determined the enzyme concentration that would give a high enough polarization signal to measure binding affinities. The inhibitor constants (*K_i_*) were calculated with Equation 1 (61),

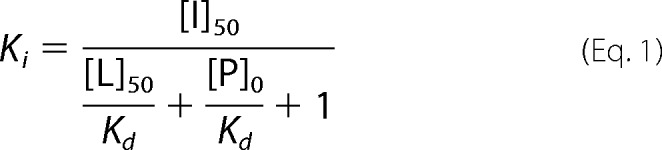
 where [I]_50_ is the concentration of the unlabeled compound at 50% inhibition, [L]_50_ is the concentration of the free probe-cyclophilin enzyme at 50% inhibition, [P]_0_ is the concentration of the free protein at 0% inhibition, and *K_d_* is the dissociation constant of the probe-protein complex.

Assays were conducted in 384-black low flange non-binding microtiter plates (Corning, Inc.). A total solution of 80 μl was used consisting of three components: fluorescent cyclosporine probe (FP-CsA) (45 nm), enzyme (40 nm), and inhibitor (10–10000 nm). At least three replicates were used for each experiment. The percentage of DMSO in the total solution should remain lower than 1%. The FP probe, enzyme, inhibitor, and FP-CsA were incubated in the OmegaFluostar at room temperature for 30 min. FP-CsA (40 μl) was added last. Measurements were taken after gain adjustment of control sample and 30-min incubation with a xenon flash light with filter settings for 485-nm excitation and 520-nm emission.

##### Mitochondrial Isolation

Subcellular fractionation was performed as described previously (62). Briefly, C57BL/6J WT or CypD^−/−^ (63) male mice of 3–6 months were sacrificed by cervical dislocation, and their liver was removed and placed immediately into ice-cold isolation buffer (250 mm mannitol, 5 mm HEPES, 0.5 mm EGTA, pH 7.4). At 4 °C, the liver was rinsed in PBS to remove excess blood, and any fat and connective tissue was eliminated. PBS was then replaced with isolation buffer containing 1 mm PMSF, and the liver was chopped into pieces (∼2 mm in length). Tissue was then homogenized in this solution until no solid matter remained and then centrifuged at 800 × *g* for 10 min at 4 °C. The nuclear pellet was then discarded, and the postnuclear supernatant was retained and centrifuged at 10,300 × *g* for another 10 min at 4 °C. The postmitochondrial supernatant was discarded, and the mitochondrial pellet was resuspended in isolation buffer and PMSF and kept on ice. Protein levels were quantified using a Thermo Scientific BCA protein quantification assay, as per the manufacturer's instructions.

##### Calcium Retention Capacity Assay

Isolated mitochondria were resuspended (500 μg of protein/ml) in MSK buffer (75 mm mannitol, 25 mm sucrose, 5 mm potassium phosphate monobasic, 20 mm Tris-HCl, 100 mm KCl, and 0.1% bovine serum albumin, pH 7.4) supplemented with 10 mm succinate, 1 μm rotenone, and 1 μm Fluo5N. 200 μl of mitochondrial suspension per well was used in 96-well microplates. Compounds were incubated for 10 min before the plate was assayed in a Fluostar Optima plate reader, using excitation/emission filters at 480/520 nm; CaCl_2_ was injected approximately every 6.5 min for 80 min (12 total injections, final concentration of 75 μm). To calculate percentage inhibition of Ca^2+^-induced pore opening, first areas under each curve were calculated, and controls without CaCl_2_ addition were subtracted as background. The background-corrected values were then expressed as the fraction of controls without mitochondria, representing the total amount of Ca^2+^ added, unbuffered by mitochondria. Percentage inhibition for each [compound] was then calculated as the percentage of the corresponding value for the untreated condition. Significance was assessed by one-way ANOVA, in comparison with CsA control. For experiments with CypD^−/−^ mice, 100 μl of mitochondrial suspension per well was used. CaCl_2_ was injected approximately every 6.5 min for 135 min (20 total injections, final concentration of 266 μm). Data were background-corrected and expressed as the fraction of controls without mitochondria and then normalized to the wild type no drug condition. Significance was assessed by one-way ANOVA.

##### Respirometry

Oxygen consumption was measured using Oroboros Oxygraph-2K as described previously (62). Prior to the assay, the Oxygraph chambers were calibrated with Miro5 buffer (0.5 mm EGTA, 3 mm MgCl_2_·6H_2_O, 60 mm potassium lactobionate, 20 mm taurine, 10 mm KH_2_PO_4_, 20 mm HEPES, 110 mm sucrose, 1 g/liter BSA (essentially fatty acid-free)). Isolated mitochondria were suspended in Miro5 (at 100–200 μg/ml) and loaded into the chamber together with substrates (malate, 2 mm; glutamate, 10 mm), and the O_2_ flow signal was allowed to stabilize to the basal respiration rate (∼10 min). Compounds were added to the chambers at the following concentrations and order: DMSO/CsA/JW47 (concentration as indicated) to produce the basal rate after compound addition (AC), ADP (2.5 mm) to give state 3 respiration, oligomycin (2.5 μm) to give leak respiration, carbonyl cyanide *p*-trifluoromethoxyphenylhydrazone (FCCP) (titrated to produce maximal respiratory capacity), and antimycin A (2.5 μm) to give non-mitochondrial respiration. Data were analyzed by subtracting the antimycin A respiration rate to give mitochondrial specific O_2_ flow and were then expressed as a percentage of the basal O_2_ flow. Significance was assessed by one-way ANOVA, in comparison with DMSO control.

##### Measurement of Mitochondrial Membrane Potential

Rat cortical neurons, cultured for 8–9 days, were incubated for 40 min at 37 °C with the cell-permeant cationic dye tetramethylrhodamine methyl ester (25 nm), and fluorescence was measured using the ImageXpress Micro XL system (Molecular Devices). Fluorescence was measured for 7 min prior to the addition of DMSO, CsA, or JW47 (both at 40 nm and 1 μm) and then for a further 50 min before the addition of the mitochondrial uncoupler FCCP (2.5 μm) as a positive control. The minimum value after the addition of compound (prior to the addition of FCCP) was taken, and this was expressed as a percentage (using the baseline as 100% and FCCP as 0%) and then normalized to DMSO (100%). Significance was assessed by one-way ANOVA, in comparison with DMSO control.

##### Measurement of Mitochondrial Membrane Potential (ex Vivo)

Freshly isolated mouse liver mitochondria were suspended in MSK buffer containing 10 μg/ml rhodamine 123 (dequench mode), at a concentration of 500 μg/ml, and plated in an opaque black 96-well plate. Baseline fluorescence was then measured every 60 s for 5 min in a Fluostar Optima (excitation 480 nm/emission 520 nm) before the manual addition of compounds (concentrations as specified). Fluorescence measurements were continued for 45 min until the addition of 2 μm FCCP, followed by a further 10 min of fluorescence readings.

##### ATP Production

Freshly isolated mitochondria were resuspended in MSK buffer (containing 10 mm glutamate and 2 mm malate) at 1 mg/ml and plated in opaque white 96-well plates, or for neuronal assays, neurons were used 9 days after plating at 15,000 cells/well. Drugs were added at the concentrations specified and, for mitochondrial assays, were incubated for 10 min before the addition of ADP (5 mm), followed by another 45 min. For neuronal assays, drugs were added in neurobasal medium and incubated for 60 min. Cell Titer Glo reagent was then added, and the plate was shaken for 2 min in the dark to lyse cells/mitochondria and release ATP. The plates were incubated a further 10 min, and then luminescence values were read using an Optima FluoStar. ATP production data were normalized to DMSO control, and significance was assessed by one-way ANOVA.

##### Cytotoxicity in HepG2 Cells

HepG2 cells were seeded in black, clear-bottom 96-well tissue culture plates at a density of 3000 cells/well. The cells were incubated for 24 h in culture medium and then exposed (in three replicates) to increasing doses of test compound or to vehicle control (0.5% DMSO). The cells were exposed for 72 h before running the high content screening assays. The high content screening assay was multiplexed to determine mitochondrial membrane potential and mitochondrial mass using MitoTracker® (Life Technologies), cytochrome *c* release (antibody, Abcam), and membrane permeability (YO-PRO^TM^-1, Life Technologies). Cell count, nuclear size, and DNA structure were also measured (Hoechst 33342, Life Technologies). Following staining of the HepG2 cells, fluorescence was analyzed by image acquisition with a Thermo Fisher Cellomics® ArrayScanVTI High Content Screening Reader (Thermo Fisher Scientific) and vHCS^TM^view software (Thermo Fisher Scientific). 20 fields were imaged per well using a ×10 wide field objective. The image acquisition data were normalized to vehicle control values. Dose-response curves were defined and evaluated with the following equations,

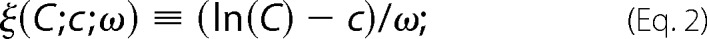


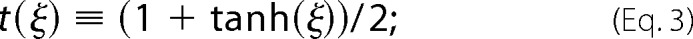


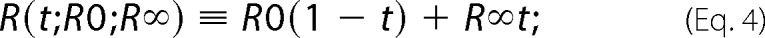
 in which *C* represents the test compound concentration, and *R*0, *R*∞, *c*, and ω are fitting parameters. The final response at a given concentration *C* is expressed as *R*(*t*(ξ(*C*; *c*; ω));*R*0;*R*∞). It was restricted such that ω > 0, which implies *R* → *R*0 as *C* → 0 and *R* → *R*∞ as *C* → ∞. The coefficient of determination (*R*^2^) was calculated for each compound and dose-response curve. An *R*^2^ value of >0.65 was used as quality control criteria and was required in all response curves.

##### Cell-based Assay for CypA Activity

VSV-G pseudotyped GFP-encoding HIV-1 vector was prepared by triple plasmid transfection of 293T cells with Fugene 6 (Roche Applied Science) as follows. Confluent 293T cells in a 10-cm dish were transfected with a mixture of 10 μl of Fugene-6 in 200 μl of Opti-MEM (Gibco) with 1 μg of pMDG VSV-G expression vector (64), 1 μg of p8.91 HIV-1 gag-pol expression vector (65), and 1.5 μg of lentiviral expression vector encoding enhanced GFP protein, CSGW (66). Viral supernatant was collected 48 h post-transfection and stored at −80 °C.

To generate CRFK cells stably expressing N-terminally HA-tagged TRIM-CypA from an EXN-based vector, murine leukemia virus (MLV) vector was prepared as above, using pMDG, CMVi MLV gag-pol expression vector, and γ-retroviral expression vector encoding a fusion protein comprising human CypA downstream of owl monkey TRIM5 RBCC (EXN-TRIM-CypA) (67). CRFK cells, which are null for TRIM5α activity (68), were then transduced with vector, followed by selection of cells in 1 mg/ml G418 (Invitrogen).

To test for the ability of drug to rescue HIV-1 infectivity in the presence of TRIM-CypA, CRFK cells were infected with a single dose of virus that infected around 20% of the cells, in the presence of DMSO, CsA (0.3–10 μm), or JW47 (0.6–20 μm). Infectivity was measured by flow cytometry 48 h postinfection.

##### P-glycoprotein Activity

Assessment of drug transporter activity was made using the Pgp-Glo^TM^ assay (Promega, Madison, WI) containing recombinant human ABCB1 in membranes and according to the manufacturer's instructions. Briefly, samples were preincubated with ATP before incubation with 100 mm compound or positive control. The residual ATP was assayed by luciferin bioluminescence.

##### In Vitro Mitogenic T Cell Stimulation

Spleens were isolated from ABH mice, and tissue was homogenized through a cell strainer (BD Biosciences) into Dulbecco's modified Eagle's medium (DMEM; Invitrogen, Paisley, UK) containing 10% fetal calf serum (FCS; Gibco, Invitrogen), 2 mm l-glutamine (Invitrogen), 100 units/ml penicillin, 100 μg/ml streptomycin (Invitrogen), and 50 μm 2-mercaptoethanol (Invitrogen). Cells were centrifuged at 500 × *g* for 5 min, and erythrocytes were lysed using 0.87% ammonium chloride following incubation for 5 min at 37 °C. Cells were washed, and viable cells were counted using trypan blue (Sigma-Aldrich) exclusion. 4 × 10^−5^ cells/well were incubated in 96-well microtest U-bottom plates (Falcon BD, Oxford, UK) in a final volume of 200 μl of DMEM. Cells were incubated with either 10-fold dilutions (range 10 nm to 10 μm) of CsA (Sandoz, Basel, CH) or JW47 diluted in DMEM from a 50 mm stock in DMSO. Cells were incubated with either 5 μg/ml concanavalin A (Sigma-Aldrich) mitogen or 0.5 μg/ml mitogenic mouse CD3 and mouse CD28-specific antibodies (Pharmingen, Oxford, UK). The cells were incubated at 37 °C for 18–22 h before the addition of 1 μCi of [^3^H]thymidine (PerkinElmer Life Sciences)/well. After additional incubation for 16–20 h, the 96-well plates (Microtest U-bottom, Falcon BD) were harvested (Harvester 96, Mach III M, TOMTEC) onto glass fiber filters (PerkinElmer Life Sciences). After drying, a scintillation sheet (MeltiLexA, PerkinElmer Life Sciences) was melted onto the filter using a hot plate (RET Basic, IKA, Staufen, Germany). Samples were analyzed using scintillation counting (MicroBeta Plus liquid scintillation counter, PerkinElmer Life Sciences), and [^3^H]thymidine incorporation was assessed in at least triplicate samples.

##### Myelin Antigen-induced T Cell Proliferation

ABH mice were injected subcutaneously in the flank with 100 μg of myelin oligodendrocyte glycoprotein (MOG) peptide residues 35–55 (Cambridge Research Biochemicals Ltd., Billingham, UK) emulsified in Freund's adjuvant containing 200 μg of *Mycobacterium tuberculosis* H37Ra (Difco, Becton Dickinson, Oxford, UK) on days 0 and 7 (69). Spleens were collected and prepared and analyzed as above except that mitogens were replaced with 5 μg/ml MOG 35–55 peptide, and cells were incubated for 72 h before the addition of tritiated thymidine.

##### Pharmacokinetic Analysis

ABH mice (*n* = 4) were injected intraperitoneally with 0.1 ml of 10 mg/kg JW47. Animals were killed 2 and 4 h later with a CO_2_ overdose, and blood was immediately collected from the heart following death. Blood was then added to Microtainer (BD Biosciences) tubes and centrifuged using an Eppendorf microcentrifuge, and plasma was collected. Following the removal of blood, the brain was rapidly (<30 s) dissected from the skull and stored at −80 °C prior to analysis by a contract research organization using liquid crystal mass spectroscopy.

##### In Vivo T Cell Proliferation

The contact sensitizer 4-ethoxymethylene-2-phenyl-2-oxazolin-5-one (oxazolone; Sigma) was dissolved (25 mg/ml) in 4:1 acetone/olive oil. Mice (*n* = 3/group) received epicutaneous application of either 25 μl of 2.5% oxazolone or acetone/olive oil on the dorsum of the ear on day 0 (70). The draining auricular lymph nodes were removed 3 days later, and the induced proliferative response was assessed as described previously. Briefly, 5 × 10^5^ cells/well were cultured in RPMI 1640 medium with glutamate (Gibco®, Invitrogen Ltd., Paisley UK), supplemented with 0.5 mm sodium, in round-bottomed 96-well plates overnight at 37 °C in a humidified atmosphere of 5% CO_2_ in the presence of 1 μCi of [^3^H]thymidine (PerkinElmer Life Sciences) per well. DNA synthesis was estimated using β-scintillation counting as above. Animals received daily intraperitoneal injections of either vehicle or JW47 from day 0 to day 3 (14, 70). Results are expressed as mean ± S.E. thymidine incorporation counts/min.

##### Induction of Relapsing-Progressive EAE

Mice were injected subcutaneously with 1 mg of freeze-dried mouse spinal cord homogenate (SCH) in Freund's adjuvant on days 0 and 7 as described previously (71). After the initial paralytic disease and subsequent remission, a relapse was induced by a further injection of SCH in Freund's incomplete adjuvant on day 28 to induce a relapse 7 days later (71). Studies were randomized, blinded, and powered as described previously (71). Neurological scores were graded as 0 = normal, 1 = limp tail, 2 = impaired righting reflex, 3 = hind limb paresis, 4 = complete hind limb paralysis, and 5 = moribund/death (71). Results are expressed as mean ± S.E. maximum or minimum neurological score and mean day of onset ± S.D. The clinical scores are presented as the mean daily neurological score ± S.E. Differences in clinical scores were assessed using non-parametric, Mann-Whitney U statistics (71). Motor control and coordination were assessed on an accelerating (4–40 rpm, accelerating at 6 rpm/25 s) RotaRod (ENV-575M, Med Associates Inc., St. Albans, VT) as described previously (71). This was performed 1 day before induction of relapse and at the termination of the experiment on day 45. RotaRod assessment was performed blinded to treatment. Animals were randomized to vehicle or treatment based on their RotaRod scores. Results are expressed as mean ± S.E. time that animals maintained rotarod activity. Differences in rota activity and quantitative neurofilament ELISA were assessed using Student's *t* test incorporating tests for equality of variance using Sigmaplot (Systat Software, Inc., San Jose, CA) (71).

At the end of the experiment, the spinal cord was removed, and an ELISA for heavy chain neurofilament on spinal cord was performed. Total nerve content of each spinal cord was estimated following calibration against neurofilament protein standards as described previously (71, 72).

##### Neurofilament ELISA

Neurofilament level as a validated correlate of spinal cord axonal content as determined by histology was determined as follows. Spinal cords were collected from the spinal columns of untreated (*n* = 11) and JW47 (1 mg/kg)-treated (*n* = 13) animals at the second remission phase of disease postrelapse at day 45 after disease induction. Tissues were snap-frozen and stored at −80 °C before homogenization. Tissues were homogenized in a glass homogenizer in 1 ml/100 mg of spinal cord tissue wet weight homogenization buffer (0.2 mm PMSF, 1 mm EDTA, 1 mm EGTA, 4 m urea, 10 mm Tris-HCl (Sigma), pH 7.2) plus 1:100 HALT protease inhibitor mixture (Thermo Fisher Scientific) and further homogenized by sonication twice for 10 s (Cole-Parmer Instruments, Vernon Hills, IL). Samples were spun down at 13,000 rpm in a bench top centrifuge (Eppendorf), and the supernatant was collected and stored at −80 °C before neurofilament determination. Samples were thawed on ice, and an ELISA for heavy chain neurofilament was performed. Briefly, a 96-well plate was coated overnight at 4 °C with capture antibody (1:5000; SMI35 anti-neurofilament H, Cambridge Bioscience, Cambridge, UK) in coating buffer (0.15 m Na_2_CO_3_, 0.35 m NaHCO_3_ (Sigma), pH 9.6). Following one wash in wash buffer (150 mm NaCl, 10 mm Tris-HCl, 0.1% Tween 20 (Sigma), pH 7.5), nonspecific binding was blocked by incubation with 5% bovine serum albumin (Sigma) in wash buffer for 1 h at room temperature. Following a wash step, samples and standards (porcine neurofilament heavy chain, Chemicon International) were diluted in wash buffer with 1% bovine serum albumin and incubated on the plate for 1 h at room temperature. Following five wash steps, the detector antibody was applied (1:1000 rabbit anti-NF200, Sigma) and incubated for a further 1 h at room temperature. The plate was washed five times, and the reporter antibody was applied (1:1000 swine anti-rabbit HRP conjugate, DAKO). Following a final five washes, tetramethylbenzidine substrate (Sigma) was applied, and color production was measured on a BioTek Synergy HT plate reader at 450 nm.

The protein content of the samples was determined by a micro-BCA assay (Pierce, Thermo Fisher), and axonal neurofilament levels in each were calculated as μg of neurofilament/mg of total protein in each sample.

##### SMI32/SMI35 Ratio

A 96-well plate was coated with either SMI35 anti-phosphorylated Nf-H or SMI32 anti-non-phosphorylated Nf-H, which is a marker of axonal damage/dystrophy (Cambridge Bioscience) antibodies at a 1:5000 dilution as above. Due to the nature of the epitope, an absolute standard for SMI32-reactive neurofilaments was unavailable. Nf-H^SMI32^ was therefore presented as a proportion of total neurofilament as measured by absorbance level and corrected for total protein levels in each sample.

##### Study Approval

All animal procedures were approved by the local ethical review processes and government inspectors in accordance with the UK Animals (Experimental Procedures) Act 1986, which incorporates directive 2010/63/EU. Experimental details, including randomization, powering, and blinding, to conform with the ARRIVE (Animals in Research: Reporting *In Vivo* Experiments) guidelines have been reported previously (71).

## Results

### 

#### 

##### Design and Synthesis of a Selective CypD Inhibitor

We required a molecule with the following profile: (*a*) good CypD potency, (*b*) selectivity for CypD over other cellular cyclophilins by mitochondrial targeting, (*c*) significant brain levels indicating an ability to target MS lesions, (*d*) low immunosuppressive activity, and (*e*) a better cytotoxic profile than CsA. CsA is famously non-druglike in terms of small molecule parameters, such as Lipinksi's rules of five; however, the macrocyclic structure has been proposed to cancel out some of the non-druglike features, such as excessive hydrogen bonding (73). In chemical terms, the compound is an uncharged, 11-amino acid, macrocyclic peptide. It is lipophilic with a measured log*P* of 2.7 (74).

A ternary complex of CsA, CypA, and calcineurin is required to trigger the immunosuppressive response via inhibition of nuclear factor of activated T-cells dephosphorylation (75). Residues 3–7 are available from the Cyp-cyclosporin complex to bind calcineurin. The ternary complex is closely packed and less tolerant of changes than for Cyp binding alone. The crystal structure of SmBzCsA (Fig. 1*C*) reveals that substitution at the [Sar^3^] position does not affect cyclophilin binding but blocks calcineurin binding (76). We chose to modify CsA at the [Bmt^1^] side chain, where we predicted that substitution would have a similar negative effect on immunosuppression. Because the activation of calcineurin is also proposed to mediate some of the cytotoxic effects of CsA, we anticipated that selective molecules would improve the toxicity profile, whereas additional selectivity for CypD over the cytoplasmic cyclophilins would provide further gains.

The CsA binding cleft in cyclophilins is highly conserved, and mitochondrial targeting provides a convenient way to obtain selectivity for the mitochondrial CypD. A variety of compounds have been suggested to localize to mitochondria. Mostly, these are lipophilic cations, such as TPP^+^, but other cations are known (77). We were attracted by the possibility of utilizing the quinolinium cation as a targeting group for several reasons, including its small size and chemical simplicity. The cation is also present in dequalinium, a topical antibiotic that can also be given *in vivo* (78, 99). We utilized SmBzCsA as a non-immunosuppressive, non-targeted control (Fig. 1*B*) (36, 76). We also designed the chemical linker to be small and to be a hydrocarbon chain to minimize any effects on membrane permeability.

##### Synthesis

We employed olefin metathesis as a direct and relatively mild way to alter the natural structure of CsA. The synthesis of the [Bmt^1^]-linked CsA derivatives is outlined in Fig. 1*A*. Olefin cross-metathesis using the Grubbs-Hoyeda second generation catalyst and the quinolinium-substituted olefin yields the target analogue JW47. The synthesis of the required quinolinium olefin is also shown. Shorter linkers did not provide effective couplings in the metathesis reaction (not shown). Synthesis of the fluorescein-PEG-CsA probe for the fluorescence polarization assay was conducted using cross-metathesis, followed by hydrolysis, and successive amide coupling steps to provide the desired compound (supplemental Fig. S1).

##### X-ray Analysis of a CypD JW47 Complex

**FIGURE 2.**
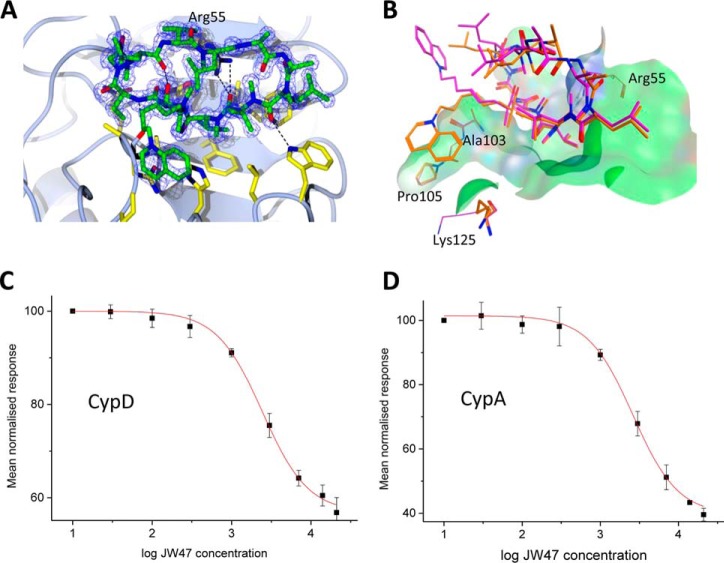
*A*, crystal structure of JW47 in complex with CypD and its effect on Ca^2+^-induced PT. Electron density of JW47 in the CypD catalytic site is shown as a *wire mesh* with JW47 modeled into the density as a *ball and stick model* (*green*). *B*, JW47 adopts two poses in the crystal structure shown in *orange* and *magenta*. The *orange pose* illustrates a possible stabilizing interaction with Ala-103, Pro-105, and Lys-125. The surface in *green* is generated from the chain that co-crystallized with this pose. Note that Lys-125 also adopts two conformations. *C*, fluorescence polarization assay for cyclophilin binding obtained using a fluorescein-labeled CsA probe. Typical data for CypD are shown. The concentration of the probe is 45 nm, and the enzyme concentration is 40 nm. Millipolarization values are fitted to a dose-response curve using Origin. *D*, fluorescence polarization data for CypA. *Error bars*, S.D.

To determine the precise binding mode of JW47 to CypD, we determined the x-ray structure of the complex to 1.1 Å resolution. We utilized the protein sequence of CypD (PPIF) with a K133I mutation, which enhances crystallization without significantly affecting activity (48). Crystals were grown with the hanging drop method, and the CypD structure Protein Data Bank entry 2Z6W was used as the search model with CsA removed. The electron density for JW47 in the active site is shown (Fig. 2*A*), with the structure of JW47 modeled in *green*. Crystallographic parameters are shown in Table 1. The core of the macrocycle is almost identical to that of CsA itself, whereas the pendant quinolinium group can adopt two poses (Fig. 2*B*). In both poses, the position of the quinolinium group is partially stabilized by crystal contacts with a symmetry-related molecule. In one of the poses, the 1-(pent-4-enyl)quinolinium (*salmon color*) extends back over the macrocycle and makes an intramolecular hydrophobic contact with [MeLeu]^4^ of JW47. In the other pose (*magenta*) the 1-(pent-4-enyl)quinolinium moiety extends in the opposite direction to lie along the surface of the CypD, making hydrophobic contacts with Ala-103, Gly-104, and Pro-105.

**TABLE 1:** **Crystallographic statistics of JW47**

Parameters	Values
Space group	P2_1_2_1_2_1_
Cell dimensions	
*a* (Å)	38.12
*b* (Å)	69.51
*c* (Å)	109.17
Matthews coefficient	2.03

**Data statistics**	
Resolution (Å)	35.99-1.08 (1.11-1.08)*^a^*
No. of measurements	1,255,737 (13,170)
No. of unique reflections	101,679 (2352)
Completeness (%)	81.5 (24.7)
*R*_meas_	0.061 (1.279)
*R*_pim_*^b^*	0.017 (0.518)
*I*/σ(*I*)	22.0 (1.4)

**Refinement statistics**	
*R*_work_ (%)*^c^*	0.0981
*R*_free_ (%)*^c^*	0.1312
No. of protein atoms	2600
No. of water atoms	304
No. of ligand atoms	194
Mean overall *B* value (Å^2^)	15.020
Data used in refinement	
Resolution range (Å)	35.99–1.25
Completeness for range	97.48
No. of reflections	75,156
Root mean square deviations from ideal geometry	
Bond lengths (Å)	0.0224
Bond angles (degrees)	2.1375
Ramachadran analysis*^d^*	
No. in favored regions (%)	316 (97.2)
No. in allowed regions (%)	9 (2.8)
Outliers	0 (0)

*^a^* Values in parentheses are for the highest resolution shell.

*^b^* Precision-indicating merging *R* factor, *R*_pim_ = Σ*_h_*(1/(*n_h_* − 1))½ Σ*_l_*|*I_hl_* − 〈*I_h_*〉|/Σ*_h_*Σ*_l_*〈I*_h_*, where *n_h_* is the number of observations of reflection *h*, *I_hl_* is the *l*th observation of reflection *h*, and 〈*I_h_*〉 is the average intensity for all observations *l* of reflection *h*.

*^c^ R*_work_ and *R*_free_ = Σ‖*F*_obs_| − |*F*_calc_‖/Σ|*F*_obs_| × 100 for 95% of the recorded data (*R*_work_) and 5% of the data (*R*_free_). *F*_obs_ is the observed structure factor amplitude, and *F*_calc_ is the calculated structure factor amplitude.

*^d^* Determined by MolProbity.

##### Cyclophilin Enzyme Binding

A fluorescence polarization assay was used to determine cyclophilin binding. CsA gives binding constants of *K_i_* = 1.4 nm for CypD and 22.5 nm for CypA. These affinities are broadly in agreement with data from other laboratories (59, 60). The CsA analogue, SmBzCsA, also demonstrated potency in this assay with *K_i_* of CypD = 236 nm and *K_i_* of CypA = 202 nm (Table 2), consistent with our data utilizing the classical chymotrypsin-based system (36). JW47 showed similar binding to SmBzCsA at *K_i_* of CypD = 202 nm and *K_i_* of CypA = 236 nm. Representative dose-response curves for CypD and CypA are shown (Fig. 2, *C* and *D*).

**TABLE 2:** **CypA and CypD binding using fluorescence polarization**

Compound	*K_i_*	
	CypA	K133I CypD
	*nm*	
CsA	22.5 ± 4.3	1.4 ± 1.6
JW47	504.5 ± 9.2	298.0 ± 17.9
SmBz	236.2 ± 8.4	202.1 ± 14.1

##### The Quinolinium Group Localizes Fluorescein to Mitochondria

To confirm that quinolinium cation can localize cargo to mitochondria, we took a membrane-impermeant dye, carboxyfluorescein, and conjugated it to quinolinium using an alkyl linker (see supplemental Methods). The linked dye diffused into cells (although some remained extracellularly) and co-localized with tetramethylrhodamine methyl ester, demonstrating the mitochondriotropic properties of quinolinium (data not shown).

##### JW47 Is a Highly Potent Inhibitor of Ca^2+^-mediated PT Pore Formation

**FIGURE 3.**
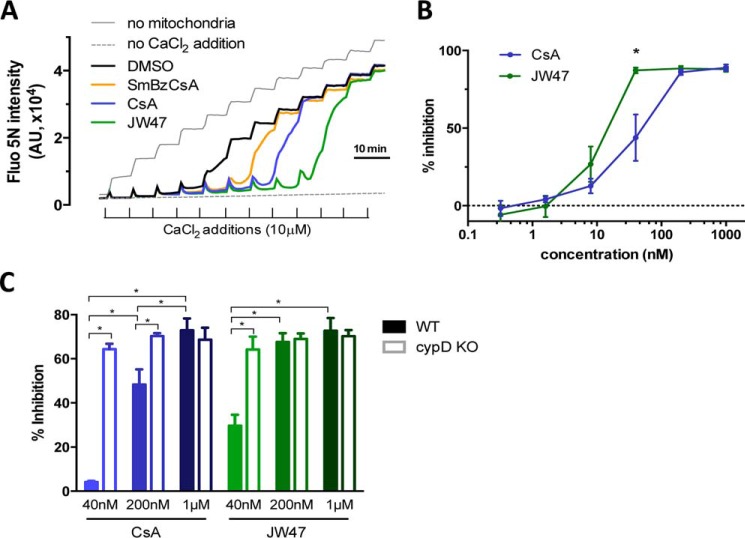
*A*, comparison of the effect of JW47, CsA, and SmBzCsA on mitochondrial CRC. Shown are representative traces of the CRC assay in isolated rat liver mitochondria. Fluo-5N fluorescence was measured in the extramitochondrial solution following repeated additions of Ca^2+^ (10 μm; for details see “Experimental Procedures”). Increase in fluorescence indicates loss of Ca^2+^ retention due to PT pore opening. Inhibition of PT pore with different compounds (added at 200 nm) increases CRC, represented by the delay in increase of fluorescence. *B*, dose-response curves of JW47 and CsA on PT inhibition (expressed as percentage increase in CRC compared with DMSO treatment). *C*, quantification of the dose-dependent effects of CsA and JW47 on CRC (PT) in liver mitochondria isolated from WT and CypD KO animals. Percentage inhibition denotes increase in CRC compared with DMSO treatment, normalized to WT. *, *p* < 0.05 (*t* test). *Error bars*, S.E.

In order to assess the efficiency of compounds on Ca^2+^-mediated PT pore formation, we measured the calcium retention capacity (CRC) of isolated mouse liver mitochondria. The Ca^2+^ concentration in the extramitochondrial solution was measured using the membrane-impermeable low affinity fluorescent Ca^2+^ sensitive dye Fluo-5N following repeated addition of Ca^2+^ boluses (10 μm). Energized mitochondria take up Ca^2+^, resulting in a declining fluorescent signal following the Ca^2+^ bolus-induced peak. Mitochondria take up and buffer Ca^2+^ up to a threshold when intramitochondrial [Ca^2+^] reaches the threshold to induce PT. This results in loss of mitochondrial membrane potential, preventing further Ca^2+^ uptake, resulting in lack of Ca^2+^ buffering, represented by a stepwise increase in extramitochondrial [Ca^2+^] at each Ca^2+^ addition (Fig. 3*A*). The amount of Ca^2+^ required to induce PT characterizes its Ca^2+^ sensitivity and defines mitochondrial CRC. Inhibition of CypD, the Ca^2+^ sensor of PT, thus leads to increased CRC. JW47 inhibited Ca^2+^-induced PT (*i.e.* increased CRC) with significantly higher potency as compared with CsA and the non-immunosuppressive inhibitor SmBzCsA. JW47 showed half-maximal inhibition at ∼10 nm as compared with ∼40 nm for CsA in the CRC assay (Fig. 3*B*). These results show that JW47 is an approximately 4-fold more potent inhibitor of Ca^2+^-mediated PT pore opening than CsA. In order to confirm that JW47 selectively targets CypD to reduce Ca^2+^ sensitivity of PT pore formation, the efficiency of the compound was tested on mitochondria isolated from CypD knock-out mice. Neither CsA nor JW47 had any effect on CRC from CypD KO mice (Fig. 3*C*), whereas CRC in the mitochondria from WT mice was significantly increased by both compounds, proving that JW47 inhibits PT pore opening via binding to CypD.

##### JW47 Has a Wide Safety Window over Effects on Mitochondrial Membrane Potential or Oxidative Phosphorylation

**FIGURE 4.**
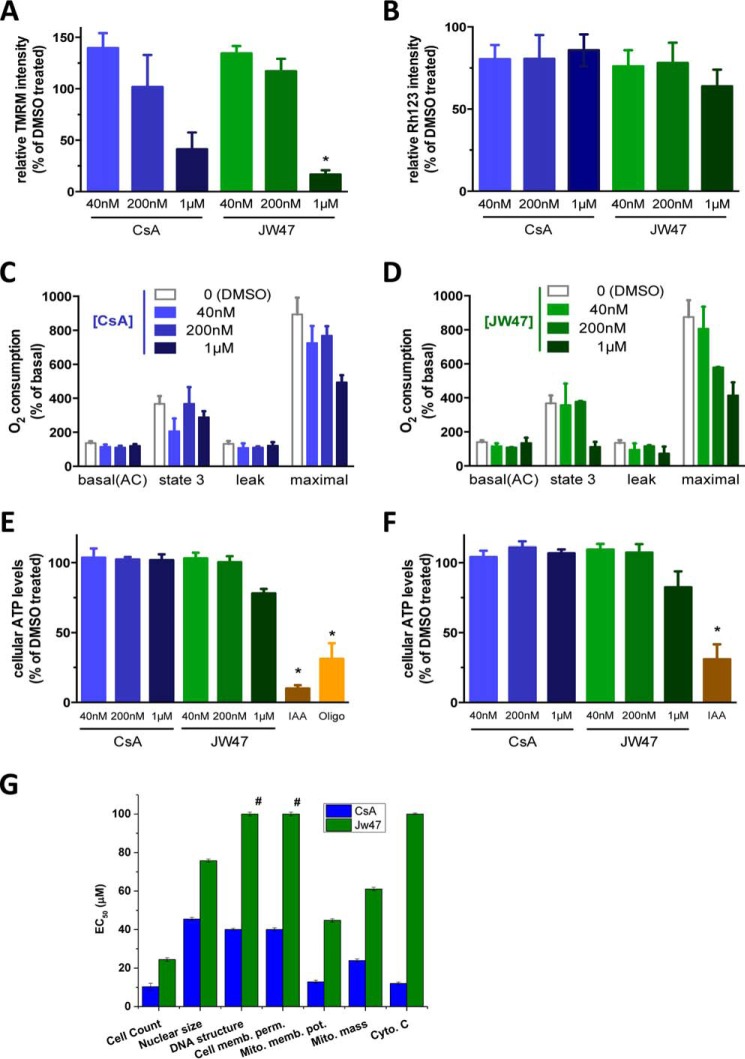
**Mitochondrial and cellular toxicological assessment of JW47 and CsA.** Mitochondrial parameters were measured in rat cortical neurons (*A* and *E*) and isolated rat liver mitochondria (*B–D* and *F*). *A* and *B*, mitochondrial membrane potential was measured in tetramethylrhodamine methyl ester (*TMRM*)-loaded neurons using ImageXpress MicroXL (*A*) and in rhodamine-123 loaded isolated mitochondria using a fluorescent plate reader (*B*). Values are normalized to DMSO (100%)- and FCCP (2.5 μm, 0%)-treated samples. *, *p* < 0.05 (one-way ANOVA). *C* and *D*, O_2_ consumption was measured in mitochondria isolated from rat liver in the presence of glutamate and malate using an Oroboros high resolution oxygraph as described under “Experimental Procedures.” The effect of compounds on basal, leak (oligomycin, 2.5 μm), and maximal uncoupled respiration (FCCP, titrated to give maximum effect) is shown, as compared with basal, DMSO controls. *, *p* < 0.05 (paired *t* tests). *E* and *F*, ATP levels in cortical neurons (*E*) and ATP production of isolated mitochondria in the presence of substrates and ADP (*F*) was measured using a luciferase assay as described under “Experimental Procedures.” Iodoacetic acid (*IAA*; 1 mm) and oligomycin (*oligo*; 2.5 μm) were used to show the contribution of glycolysis and mitochondrial ATP synthesis, respectively. *, *p* < 0.05 (*t* test). *G*, *in vitro* toxicological assessment of CsA and JW47. HepG2 cells were plated, and after 24 h, the cells were treated with the compounds at a range of concentrations. At the end of the incubation period, the cells were loaded with the relevant dye/antibody for each cell health marker and scanned (see “Experimental Procedures”). Data are shown as EC_50_ values (μm) ± *R*^2^. #, no response observed at 100 μm. *Error bars*, S.E.

To assess the potential adverse effects of JW47, we measured fundamental mitochondrial functional parameters both in cultured rat neurons and in isolated mitochondria and compared the effects of CsA and JW47 above concentrations causing maximal inhibition of the PT pore (>200 and 40 nm, respectively). Neither mitochondrial membrane potential (Fig. 4, *A* and *B*), oxygen consumption (Fig. 4, *C* and *D*), nor ATP production (Fig. 4, *E* and *F*) were affected by supramaximal JW47 (up to 200 nm) or CsA (up to 1 μm) in either models. JW47 inhibited neuronal mitochondrial membrane potential only at ∼25 times higher concentrations (1 μm), than the concentration which produced maximal inhibitory effect (40 nm) on the PT pore.

##### Cytotoxicity in HepG2 Cells

HepG2 cells represent a convenient model for exploring hepatotoxicity and can be used to estimate multiple cell health parameters simultaneously. JW47 showed less toxicity in all measures examined, most notably cytochrome *c* release (Fig. 4*G*).

##### Estimation of Cellular CypA Activity Using an HIV-based Cellular Assay

**FIGURE 5.**
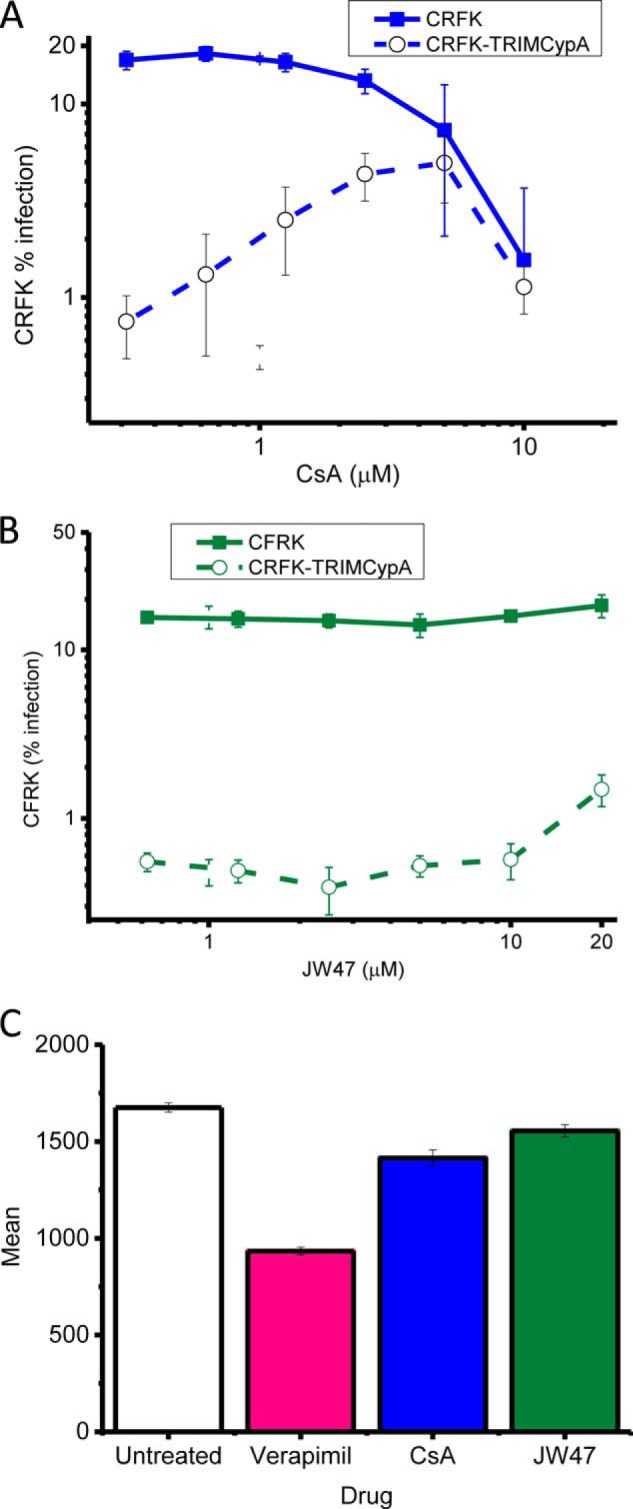
**Assessment of in-cell CypA binding.** CRFK cells transduced with either empty vector (*filled squares*) or TRIM-CypA (*open circles*) were infected with VSV-pseudotyped GFP-expressing HIV-1 vector in the presence of DMSO or increasing concentration of drug. *A*, CsA; *B*, JW47. Viral infection was measured by flow cytometry at 48 h postinfection. Data are the average of three independent experiments. *C*, P-glycoprotein drug transporter activity. Vehicle, verapamil (100 μm), and CsA analogues were tested as substrates for P-glycoprotein (Pgp-Glo assay). Results are the mean ± S.E. (*error bars*) bioluminescence measurements from the luciferin reporter.

To test cellular cyclophilin selectivity of JW47, we conducted an HIV-1-based cellular assay responsive to CypA inhibition. HIV-1 infection of cell lines can be inhibited by the expression of an artificial antiviral protein, comprising the RBCC domains of owl monkey tripartite motif-containing protein 5 (TRIM5) fused to human CypA (TRIM-CypA). TRIM-CypA inhibited viral infection by 32-fold in the absence of drug (Fig. 5*A*). CsA rescued infectivity through CypA inhibition, whereas JW47 rescued infectivity poorly and only at concentrations of >10 μm (Fig. 5*B*). A drop to infectivity in non-restricting cells was due to drug toxicity at 5 μm CsA and above. JW47 showed no evidence for toxicity at any of the concentrations tested.

##### Activity against Drug and Anion Transporters

The ability of the compounds to affect human P-glycoprotein (ABCB1) drug transporter activity was assessed using a bioluminescence assay. The standard inhibitor verapamil at 100 μm showed a 40% reduction in luminescence (Fig. 5*C*). Despite being noted as a P-glycoprotein inhibitor (79), CsA showed only a 16% inhibition at 100 μm. JW47 showed less inhibition than CsA, 8% at 100 μm (Fig. 5*C*). Activity against organic anion transporters OATP1B1 and OATP1B3 has been linked to drug-induced unconjugated hyperbilirubinemia in patients receiving alisoporivir (80). JW47 demonstrated 7–8-fold less inhibition of β-estradiol-17-β-d-glucuronide uptake for the OATP1B1 transporter than CsA (2.56 ± 0.47 μm *versus* 0.44 ± 0.084 μm for CsA). Similarly, for the OATP1B3 transporter, JW47 showed 4-fold less activity than CsA for inhibition of CCK-8 transport (IC_50_ = 0.20 ± 0.11 μm *versus* 0.82 ± 0.07 μm).

##### Pharmacokinetics

JW47 pharmacokinetics was determined in normal ABH mice at 10 mg/kg intraperitoneally at 2 and 4 h. JW47 showed high plasma levels of 10.1 μm at 2 h (Table 3) and appreciable although much lower brain levels (13.2 nm). A 10 mg/kg dose was used to enable detection in the brain. This is broadly comparable with CsA in rodents (79).

**TABLE 3:** **Plasma and brain levels of Jw47 in ABH mice following a single 10 mg/kg dose**

Time	Plasma		Brain	
	μg/ml	μm	μg/g	nm
*h*				
2	13.74 ± 3.84	10.1	0.018 ± 0.0019	13.2
4	4.90 ± 0.85	3.60	0.017 ± 0.0016	12.5

##### JW47 Is Markedly Less Immunosuppressive than CsA

**FIGURE 6.**
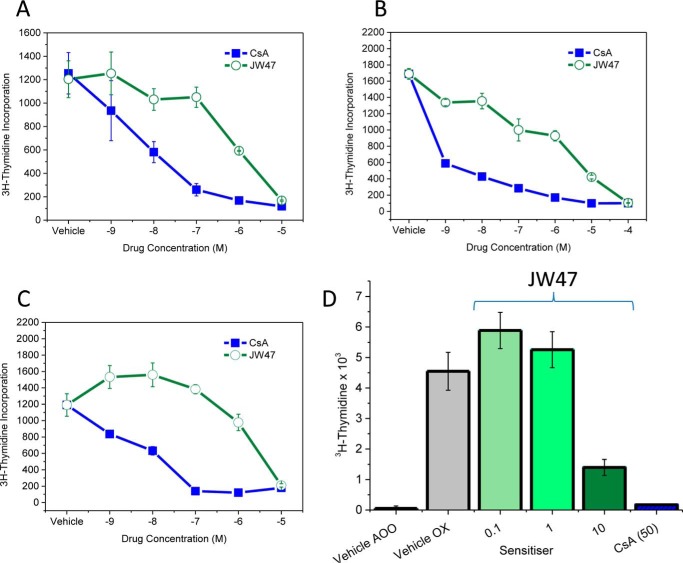
**The oxazolone contact hypersensitivity test.** A low severity *in vivo* measure of T cell proliferation is used as a rapid screen for immunosuppressive doses of agents. Oxazolone administered to the ear induces an increase in cell number and proliferation in the draining auricular lymph node. *Error bars*, S.E.

The inhibitory effect of JW47 on T cell responses was examined *in vitro*. Concanavalin A and mitogenic CD3/CD28 monoclonal antibodies induce mitogenic T cell proliferative responses that were inhibited by CsA typically in the 1–10 nm range (Fig. 6, *A–C*). JW47 only exhibited marked immunomodulation in the 1–10 μm range and was cytopathic at 100 μm. Similarly, JW47 exhibited markedly less immunosuppressive activity compared with CsA in an antigen-driven proliferation assay using myelin peptide (myelin oligoglycoprotein residues 35–55) antigen-induced T cell proliferation.

**FIGURE 7.**
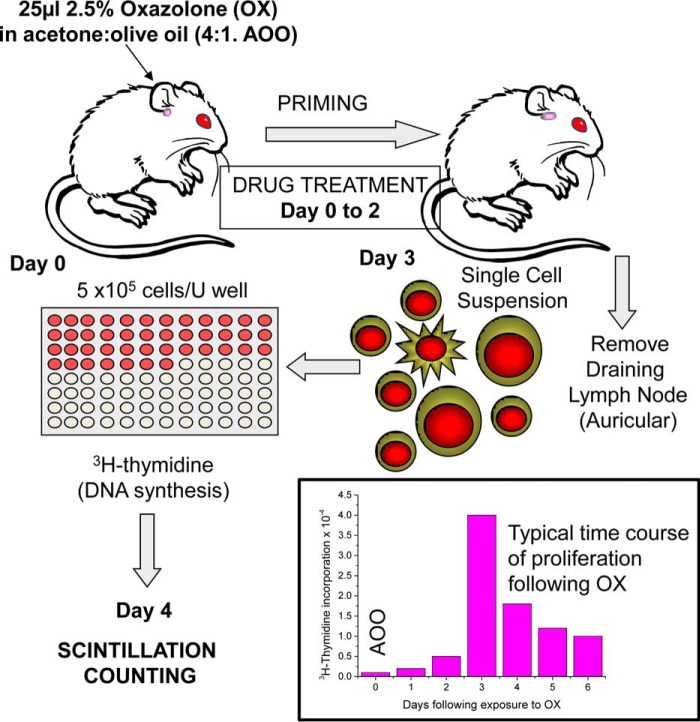
**JW47 exhibits less immunosuppressive activity than CsA.** Mitogenesis *in vitro* of normal mouse splenocyte cells is shown. Splenocytes were incubated with 5 μg/ml concanavalin A (*A*), mitogenic CD3/CD28 monoclonal antibodies (*B*), or splenocytes from MOG residue 35–55 peptide-immunized mice (*C*) in the presence of 5 μg/ml MOG peptide with vehicle or compounds for 2 (*A* and *B*) or 4 (*C*) days prior to the addition of 1 μCi of [^3^H]thymidine and were harvested 16–20 h later, and tritiated thymidine incorporation was assessed by β-scintillation counting. The results represent the mean ± S.E. of triplicate samples. *D*, low doses of JW47 *in vivo* exhibited no immunosuppressive activity. 25 μl of 2.5% oxazolone (*OX*) or acetone/olive oil (4:1) vehicle (*AOO*) was applied to the ear skin of ABH mice on day 0. On day 3, the draining auricular lymph nodes of 3–4 mice/group were removed, and 5 × 10^5^ cells were cultured overnight in the presence of 1 μCi of [^3^H]thymidine. Animals were treated with 0.1 ml of vehicle, 0.1–10 mg/kg JW47, or 50 mg/kg CsA. The results represent the mean ± S.E. of at least quadruplicate samples.

The dominant pathogenic antigen in spinal cord homogenate-induced disease in ABH mice is a hydrophobic residue in proteolipid protein that does not produce robust T cell proliferative responses *in vitro*. Therefore, to identify non-immunosuppressive doses of potential neuroprotective compounds for use in models of MS (14, 70), we employed a model using epicutaneous application of the ear skin sensitizer, oxazolone, to induce a T cell proliferative response in the draining auricular lymph node peaking 3 days later (Fig. 7) (70). Dose response of JW47 in this contact hypersensitivity model showed that daily injection of 1 and 0.1 mg/kg intraperitoneally had no effect, whereas 10 mg/kg intraperitoneally inhibited the T cell response. CsA was immunosuppressive (Fig. 6*D*) at doses known to inhibit T cell proliferation and EAE (70). Daily dosing of 1 mg/kg intraperitoneal JW47 was therefore chosen as a non-immunosuppressive dose for *in vivo* studies.

##### JW47 Induces Neuroprotection in Vivo and Slows the Accumulation of Disability in an Experimental Model of Multiple Sclerosis

**FIGURE 8.**
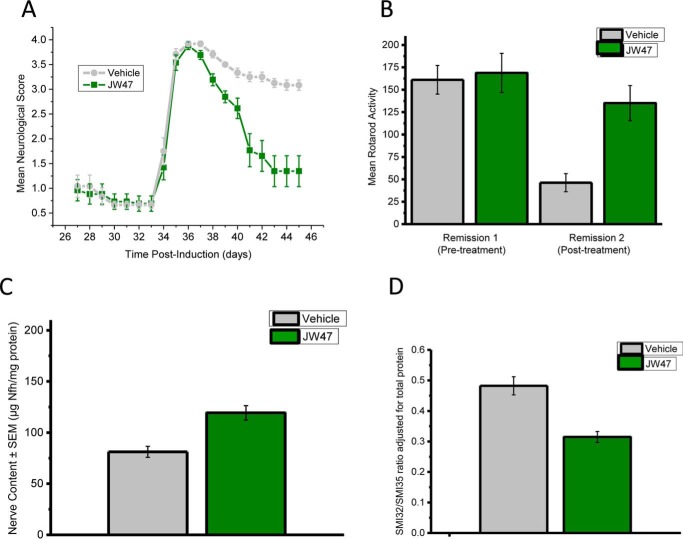
**JW47 limits the accumulation of neurodegeneration and disability during relapsing autoimmune encephalomyelitis.** ABH mice were injected with SCH in Freund's complete adjuvant on days 0 and 7 to induce paralytic EAE. A relapse was induced on day 28 during the first remission, using a further injection of SCH in Freund's incomplete adjuvant. Animals were treated daily from day 33 onward with either 1 mg/kg intraperitoneal JW47 (*n* = 14) in ethanol/cremophor/PBS (1:1:18) or vehicle (*n* = 12). The results represent the mean daily neurological score ± S.E. (*error bars*) (*A*) and the mean rotarod activity representing the mean ± S.E. time before falling/failing to stay on an accelerating rotarod before (on day 27) or after (on day 45) treatment with either vehicle or JW47 (*B*). *** *p* < 0.001 compared with vehicle treatment. *C*, axonal content in the spinal cord following treatment of relapsing EAE with JW47 1 mg/kg measured as neurofilament level adjusted for total protein content. EAE was induced with spinal cord homogenate in complete Freund's adjuvant on days 0 and 7, and a relapse was induced by reimmunization with spinal cord homogenate in complete Freund's adjuvant at day 28. Animals were randomized according to RotaRod performance score at day 27 to receive either vehicle (cremophor (Sigma)/alcohol/phosphate-buffered saline, 1:1:18) or 1 mg/kg intraperitoneal JW47 from day 33 postinfection just prior to the development of relapse at day 35 until day 47. Animals were killed, and the spinal cords were removed using hydrostatic pressure, and axonal content was measured using a quantitative neurofilament-specific ELISA. *n* = 11 untreated, *n* = 13 JW47-treated. Shown is the ratio of dephosphorylated (SMI32-reactive) neurofilament to hyperphosphorylated (SMI35-reactive) neurofilament as measured by ELISA in spinal cord homogenates from postrelapse untreated animals (*n* = 11) or JW47 (1 mg/kg)-treated animals (*n* = 13); ***, *p* < 0.001 adjusted for total protein level.

The EAE model of multiple sclerosis can be used to assess both inflammatory and neurodegenerative aspects of the disease (81). It has been shown previously that the severity of neurological disease during disease worsening, associated with weight loss, is directly correlated to the degree of immune infiltration into the spinal cord (81–83). Immunosuppression that inhibits the incidence or severity of disease is usually associated with a reduction in the histological detection of infiltrates, demyelination, and axonal loss (81–83). Clinical disease was assessed by scoring neurological signs. In addition to this subjective read-out, objective motor outcomes were detected by assessing loss of motor coordination on an accelerating rotorod, which has been shown previously to exhibit a strong positive correlation with spinal nerve content (71). JW47 was administered daily at 1 mg/kg intraperitoneally shortly before the anticipated onset of relapse. This failed to inhibit the development or the severity of relapse, which would occur with a T cell immunosuppressive agent (84), and limited the accumulation of disability due to the inflammatory penumbra (Fig. 8*A*). Thus, there were no differences in the incidence of disease (14 of 14 for JW47 *versus* 12 of 12 for vehicle), the onset of induced relapse following injection of SCH in Freund's adjuvant on day 28 (34.5 ± 0.7 days for JW47 *versus* 34.1 ± 0.8 days for vehicle), or the maximal severity disease score (3.9 ± 0.1 for JW47 *versus* 3.9 ± 0.1 for vehicle). Animals relapsing during treatment with daily 50 mg/kg CsA developed clinical disease with a score of 3.0 ± 0.4 (*n* = 7), which is significantly (*p* < 0.05) lower than found with either vehicle or JW47 treatment and is indicative of immunosuppression. Treatment with an immunosuppressive agent, such as 250 μg of CD4-depleting (YTS191) monoclonal antibody or other immunosuppressive agents, which are optimized for treatment at this late stage, will completely eliminate relapse (70). Therefore, there was no clinical evidence that JW47 was inducing overt immunosuppression in this paradigm. However, although the minimal clinical scores during the first remission before treatment were not different (0.6 ± 0.1 JW47 *versus* 0.6 ± 0.1 vehicle), following induced relapse, JW47-treated animals accumulated less deficit as a consequence of the attack and demonstrated a significantly (*p* < 0.001) better recovery during the second remission (minimal neurological score 1.4 ± 0.3 for JW47 *versus* 3.1 ± 0.1 for vehicle (*p* < 0.001). This subjective outcome was supported by objective rotarod activity outcomes (Fig. 8*B*). Animals exhibited comparable rotorod activity on day 27 during the first remission (168.8 ± 21.8 s for JW47 *versus* 161.1 ± 16.0 s for vehicle), but there was significantly (*p* < 0.001) less loss of motor coordination following treatment with JW47 (Fig. 8*B*). During the second remission after relapse, JW47-treated animals maintained activity on an accelerating rotorod for 135.0 ± 42.9 s compared with only 46.3 ± 10.1 s in vehicle-treated animals. This activity strongly correlates with spinal nerve content in this assay (71), and it was found that JW47 lost significantly (*p* < 0.01) fewer nerves (Fig. 8*C*) and axons (Fig. 8*D*) within the spinal cord than vehicle-treated animals, which was measured using a quantitative neurofilament ELISA. The SMI35 antibody stains neurofilaments with high and low degrees of phosphorylation and reveals thick and thin axons. SMI32 does not stain thin axons. The degree of neurofilament phosphorylation is modulated by myelination (85), so our readout directly quantifies loss of small axons and, by inference, demyelination. This assay system has been validated against the histological detection of nerve loss during EAE (72, 83) and is a biomarker of neurodegeneration in MS. Thus, JW47 exhibits neuroprotective potential and can inhibit loss of nerves due to the inflammatory penumbra during EAE.

## Discussion

The permeability transition pore is linked to necrotic cell death but has been hitherto underexploited as a therapeutic target. The most well known modulator of the pore, CypD, is located on the inner mitochondrial membrane and is resistant to blockade by small molecules. CsA is the standard pan-cyclophilin inhibitor, but it shows no selectivity for CypD and exhibits significant cytotoxicity. We have shown previously that CsA can be targeted to mitochondria using the well known TPP^+^ cation. Unfortunately, triphenylphosphonium attached by long alkyl or PEG linkers increases the overall lipophilicity and molecular weight of the conjugate substantially and adversely affects pharmaceutical properties. In this study, we utilized quinolinium attached by a short alkyl chain as a mitochondrial targeting cation with a minimal effect on molecular weight and lipophilicity. Olefin cross-metathesis enabled functionalization of CsA using mild conditions that did not affect the sensitive functionality of the cyclic peptide (86). Using this methodology, we identified JW47 as a prototype inhibitor. In isolated mitochondria, JW47 delayed opening of the PT pore and was more active than CsA. Detailed measures of oxidative phosphorylation indicated that JW47 only affected cellular ATP production at supramaximal concentrations but not at the effective concentration (40 nm) or the lowest maximally effective concentration (200 nm). In cortical neurons, mitochondria were not affected by 40 nm JW47 but were significantly depolarized by 1 μm JW47. The greater effect of JW47 in mitochondrial Ca^2+^ buffering compared with CsA indicates that quinolinium is effective in directing the drug to mitochondria in accordance with its more negative electropotential and in agreement with the Nernst equation (87). JW47 has less intrinsic CypD binding than CsA but is a more potent PT pore inhibitor. In addition, in isolated mitochondria from CypD knock-out mice, JW47 had no effect on Ca^2+^-mediated PT, proving that its principal mechanism of action is mediated by CypD binding. In a system designed to detect restriction of HIV replication via CypA, JW47 showed no activity, in contrast to both CsA and SmBzCsA, indicating that JW47 has minimal ability to inhibit CypA in cells. Drug transporter studies on JW47 indicate less propensity to cause bilirubinemia via inhibition of the bilirubin transporter OATP1B1 in comparison with CsA itself and an analogue, alisporivir (88). A multiparameter study of JW47 in hepatocellular carcinoma-derived HepG2 cells showed less toxicity than CsA (89) with perhaps a surprising drop in mitochondrial toxicity, despite the expected sequestration of the ligand to mitochondria (88).

EAE is a T cell-mediated, autoimmune model of MS, which can develop relapsing autoimmunity and progressive neurodegeneration (20, 81, 84). Studies using transgenic mice in the EAE model have shown that the PT pore pathway can determine neurodegeneration independently of the peripheral immune response (20, 21, 90). Although CsA can prophylactically inhibit the generation of EAE, it is therapeutically less effective during spontaneous relapsing EAE and did not inhibit relapsing MS also (70, 91). Furthermore, the nephrotoxicity of CsA also limited its clinical utility in MS (39). Progressive MS is associated with the accumulation of nerve loss and disability, shows minimal response to peripheral immunosuppression, and is currently untreatable (92). It was found that doses of JW47 could be selected to induce neuroprotection without causing overt T cell immunosuppression. We chose ELISA detection of total spinal cord nerve content as a robust non-biased way to fully quantify nerve loss (and, by implication, myelin content). This protection may be via direct effects on nerves, although it is possible that JW47 also exhibited some influence on innate inflammatory cells, which are believed to drive progressive neurodegeneration. The level of efficacy of Jw47 is similar to that found previously with other neuroprotective agents in this experimental paradigm (14), which appear to have some predictive value for identifying neuroprotective agents in multiple sclerosis (14, 93). Relapsing disease was not inhibited with JW47, in contrast to the inhibition that would occur following T cell immunosuppression with drugs used to treat relapsing neuroimmunological disease (94). These current agents, however, do not stop progressive neurodegeneration (92); therefore, it is believed that drugs like JW47 should be used in combination with current immunosuppressive disease-modifying drugs to target the neurodegenerative effects that are currently not treated.

It is therefore of interest that CsA treatment showed some modest benefit in progressive MS (39) and in neuromyelitis optica, another immunomediated demyelinating disease (95). This suggests that inhibition of CypD activity in humans may offer some clinical benefit and would benefit from delivery of a CypD-selective inhibitor. We anticipate that JW47, which is more selective and less toxic in cells, would be better tolerated than CsA *in vivo*.

CsA is thought to be excluded from the CNS by adenosine-binding cassette drug transporters, such as an ABCB1, ABCC1, ABCC4, and ABCG2, which are present in the brain (14, 96, 97). JW47 exhibited limited activity as ABCB1 substrate compared with CsA and could be detected in the brain of normal animals. A pharmacokinetic experiment with JW47 showed potential for *in vivo* evaluation with high plasma levels of JW47 that are within the active *in vitro* dose range and significant although lower brain levels. Whereas brain levels may be higher at *C*_max_, it is also probable that brain levels and importantly spinal cord levels may be significantly higher in animals with active and chronic EAE, which exhibit mainly spinal cord disease, due to alterations in metabolism and blood-brain barrier breakdown that occurs in EAE and MS (11, 71, 98). Furthermore, CNS adenosine-binding cassette drug transporters, notably P-glycoprotein (ABCB1), are down-regulated from vasculature such that compounds, even ABCB1 substrates, can be selectively targeted to lesions, despite poor apparent global CNS penetration (14). Further studies to determine the oral bioavailability, pharmacokinetic/pharmacodynamics, and toxicology of JW47 and related molecules are warranted. The action of JW47 demonstrates that pharmacological inhibitors of the PT pore are neuroprotective in EAE, as predicted using genetic ablation studies of CypD (20) and P66ShcA in the PT pore pathway (21, 99), and may provide a treatment for neurodegeneration and progression of disability during relapsing-remitting and progressive MS and other neurological diseases.

In summary, we have shown that quinolinium is an effective mitochondrial targeting group, and the CsA-tagged molecule is less cytotoxic than CsA and shows little ability to interact with cytoplasmic proteins. In mitochondria, JW47 is a more potent inhibitor of pore opening than CsA and does not affect mitochondrial function at a concentration of maximal inhibition. Furthermore, JW47 demonstrates a pronounced neuroprotective effect *in vivo* and should be a useful tool for investigation of the role of the PT pore in other neurodegenerative conditions and in models of ischemia-reperfusion injury. The quinolinium cation is smaller and is more amenable to pharmaceutical optimization than TPP^+^, and we anticipate that it will become a useful addition to the toolbox of mitochondrial targeting groups.

## Author Contributions

J. W., M. I. S., and D. S. synthesized the compounds. X. S. and A. R. C. determined the x-ray structure. G. P. and D. B. did the P-glycoprotein and *in vivo* work. F. L. and F. P. did the immunoassays and *in vivo* immunosuppression studies. J. M. H. and G. P. performed the mitochondrial Ca^2+^ buffering assays and the mitochondrial functional assays, devised, supervised, and directed by G. S. and M. R. D. M. K. and J. W. did the Cyp assays. L. H. did the cellular HIV assay. D. B. and D. L. S. obtained funding for the study. M. R. D., G. S., D. B., and D. L. S. wrote initial drafts of the paper. All authors contributed to the final manuscript.

## Supplementary Material

Supplemental Data

## Footnotes

7 The abbreviations used are:

PT: permeability transition
CRC: calcium retention capacity
CypD: cyclophilin D
CypA: cyclophilin A
CsA: cyclosporin A
EAE: experimental autoimmune encephalomyelitis
FP: fluorescence polarization
MS: multiple sclerosis
TPP^+^: triphenylphosphonium
ANOVA: analysis of variance
FCCP: carbonyl cyanide *p*-trifluoromethoxyphenylhydrazone
MOG: myelin oligodendrocyte glycoprotein
oxazolone: 4-ethoxymethylene-2-phenyl-2-oxazolin-5-one
SCH: spinal cord homogenate
Sar: sarcosine
Bmt: (4*R*)-4-[(*E*)-2-butenyl]-4-methyl-l-threonine.
