# Supplementary material for: Selective Inhibition of the Mitochondrial Permeability Transition Pore Protects against Neurodegeneration in Experimental Multiple Sclerosis
Source: J Biol Chem. 2015 Dec 17;291(9):4356–73. doi: 10.1074/jbc.M115.700385 (PMC4813465; doi:10.1074/jbc.M115.700385)
Supplement: Supplemental Data [file supp_291_9_4356__index.html]

Selective inhibition of the mitochondrial permeability transition pore protects against neuro-degeneration in experimental multiple sclerosis. — Selective Inhibition of the Mitochondrial Permeability Transition Pore Protects against Neurodegeneration in Experimental Multiple Sclerosis — Mitochondrial Protection Prevents Neurodegeneration in MS — Supplemental Data 

# Selective Inhibition of the Mitochondrial Permeability Transition Pore Protects against Neurodegeneration in Experimental Multiple Sclerosis

## Supplemental Data

- Supplemental data (.pdf, 344 KB) - Allowable supplemental chemistry data including methods and schemes.
