# Supplementary material for: Selective Inhibition of the Mitochondrial Permeability Transition Pore Protects against Neurodegeneration in Experimental Multiple Sclerosis
Source: J Biol Chem. 2015 Dec 17;291(9):4356–73. doi: 10.1074/jbc.M115.700385 (PMC4813465; doi:10.1074/jbc.M115.700385)
Supplement: Supplemental Data [file 10.1074_M115.700385_jbc.M115.700385-1.pdf]

Supplementary methods

Selective inhibition of the mitochondrial permeability transition pore protects against neuro-degeneration in EAE multiple sclerosis.

Justin Warne, Gareth Pryce, Julia Hill, Xiao Shi, Felicia Lennerås, Fabiola Puentes, Maarten Kip, Laura Hilditch, Paul Walker, Michela I. Simone, A.W. Edith Chan, Greg J. Towers, Alun Coker, Michael R Duchen, Gyorgy Szabadkai, David Baker*, David L Selwood*.

Contents

Chemistry p1

Preparation of the fluorescein labelled cyclosporine (FP-CsA) p2

Preparation of the fluorescein labelled quinolinium p5

Chemistry

All commercially available solvents and reagents were used without further treatment as received unless otherwise noted. NMR spectra were measured with a Bruker DRX 500 or 600 MHz spectrometer; chemical shifts are expressed in ppm relative to TMS as an internal standard and coupling constants (J) in Hz. LC-MS spectra were obtained using a Waters ZQ2000 single quadrupole mass spectrometer with electrospray ionisation (ESI), using an analytical C4 column (Phenomenex Gemini, 50 x 3.6 mm, 5 µm) and an AB gradient of 50–95 % for B at a flow rate of 1 mL/minute, where eluent A was 0.1:5:95 formic acid/methanol/water and eluent B was 0.:5:95 formic acid/water/methanol. High resolution mass spectra were acquired on a Waters LCT time of flight mass spectrometer with electrospray ionisation (ESI) or chemical ionization (CI).

Preparation of the fluorescein labelled cyclosporine (FP-CsA).

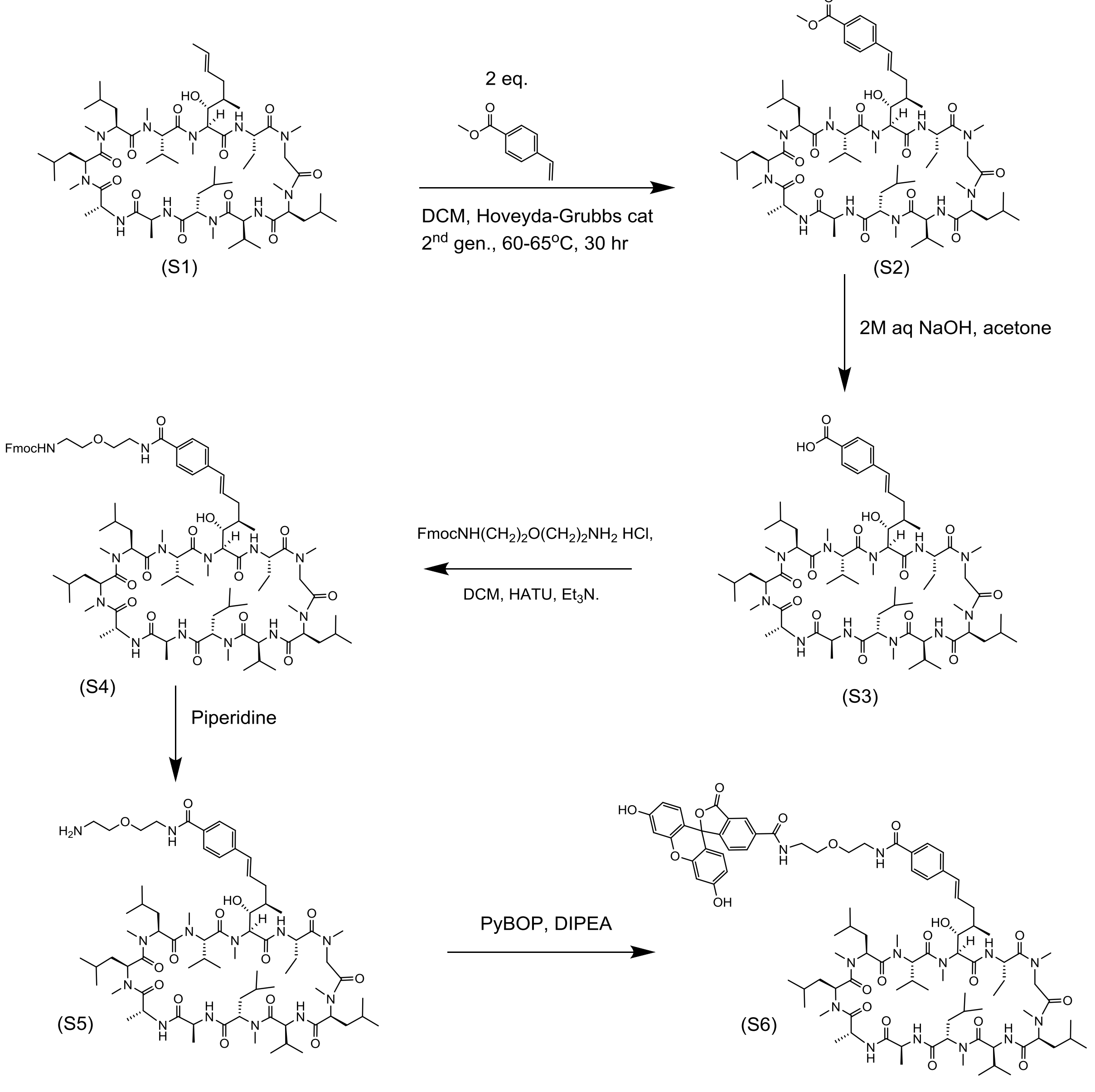

Formation of the vinyl methyl ester derivative (S2) from cyclosporine (S1).

A solution of cyclosporine (1.00 g, 0.832 mmol), methyl-4-vinylbenzoate (270 mg, 1.665 mmol) and Hoveyda-Grubbs 2nd generation catalyst (20 mg, 0.032, 4%) in dichloromethane (4 ml) was stirred at reflux (60°C) under nitrogen for 48 hours. T.l.c. analysis (acetone : cyclohexane, 1:1) of the reaction mixture showed the presence of the product (R_f 0.63) and complete consumption of the cyclosporine A starting material (R_f 0.65). LCMS analysis also confirmed the presence of the product. The reaction mixture was pre-absorbed on silica gel and purified by flash column chromatography (ethyl acetate : cyclohexane, 1:1 to ethyl acetate to ethyl acetate : methanol, 10%) and the solvent removed *in vacuo* to give a grey solid. The grey solid was then further purified by removing the Grubbs-Hoveida catalyst by letting it through an SPE-thiol column (eluant: methanol). The solvent was removed *in vacuo* to give the corresponding methyl ester as a white crystalline solid (950 mg, 86.4%).

HRMS (TOF MS ES^+): found 1344.8726 $[M+Na]^+$ $C_{69}H_{115}N_{11}O_{14}Na$ requires 1344.8523;

ν_{max} (thin film, KBr): 3466, 3418, 3318 (m-s, bumpy, CON-Hs, OH), 2961, 2935, 2873 (m, alkyl C-H), 1720 (m, conjugated C=OOMe), 1627 (s broad, bumpy, C=Os, amide I), 1520 (m broad, bumpy, C=Os, amide II) cm^{-1};

NMR δ_H ($CDCl_3$, 500 MHz): 7.92 (1H, d, *J* = 10.5 Hz, NH), 7.90 (2H, d, $J_{ArCH,ArCH}$ = 8.5 Hz, 2 x ArHs), 7.64 (1H, d, *J* = 8.0 Hz, NH), 7.47 (1H, d, *J* = 8.0 Hz, NH), 7.32 (2H, d, $J_{ArCH,ArCH}$ = 8.5 Hz, 2 x ArHs), 7.06 (1H, d, *J* = 7.5 Hz, NH), 6.34–6.24 (2H, m, alkene Hs), 5.62 (1H, dd, J = 10.5, 4.5 Hz, CαH), 5.48 (1H, d, J = 5.5 Hz, CαH, residue 1), 5.32-5.22 (2H, m, 2 x CαH), 5.07 (1H, d, J = 11.0 Hz, CαH, residue 11), 5.04-4.93 (2H, m, NHCα*H* + CαH), 4.90 (1H, dd, J = 10.0, 6.0 Hz, CαH), 4.77 (1H, app-q, J = 7.5 Hz, NHCα*H*), 4.69 (1H, d, J = 14.0 Hz, residue 3, $C\alpha H^{Re}H^{Si}$), 4.60 (1H, app-t, J = 9.0 Hz, NHCα*H*), 4.47 (1H, app-q, J = 7.5 Hz, NHCα*H*), 4.50-4.37 (1H, m, Cα*H*), 4.26-4.10 (1H, broad s, OH), 3.84 (3H, s, CO_2Me), 3.76 (1H, app-t, J = 7.0 Hz, Cβ*H*-OH, residue 1), 3.48, 3.33, 3.23 (9H, 3 x s, 3 x N-Me), 3.19-3.15 (1H, m, residue 3, $C\alpha H^{Re}H^{Si}$), 3.05, 3.04 (6H, 2 x s, 2 x N-Me), 2.70-2.65 (1H, m, obscured, Ar-CH=CH-CH^AH^B), 2.66, 2.62 (6H, 2 x s, 2 x N-Me), 1.87-1.80 (1H, m, Ar-CH=CH-CH^AH^B), 2.42-2.30, 2.10-0.60 (side-chain alkyl Hs, all residues);

NMR δ_C ($CDCl_3$, 125 MHz): 173.84 (C=O), 173.81(C=O), 173.74 (C=O), 173.50 (C=O), 171.54 (C=O), 171.17 (C=O), 170.50 (C=O), 170.44 (2 x C=O), 170.26 (C=O), 170.18 (C=O), 167.06 (C=O ester), 142.44 (Ar*Cq*-CH=CH), 132.85 (ArCq-*C*H=CH), 130.63 (ArCq-CH=*C*H), 129.88, 125.91 (2 x 2Cs, 4 x ArCs), 128.19 (*Cq*-COOMe), 75.05 (CαH-*C*H[side chain]-OH, residue 1), 58.74, 57.97, 57.56, 55.53 (2 Cs), 55.38 (6 x CαH), 51.98 (OMe ester), 50.40 ($C\alpha H_2$, residue 3), 48.85, 48.58, 48.26, 45.18 (4 x CαH), 40.50, 38.98, 37.50, 36.38, 36.02 (5Cs, 4 x CαH-CH_2-$CH(CH_3)_2$, residues 4, 6, 9 and 10; (CαH-CH[OH]-*C*H[side chain]-CH_3), residue 1), 39.46 (N-Me), 36.77 (ArCq-CH=CH-CH_2, residue 1), 34.13 (N-Me), 31.58 (N-Me), 31.36 (N-Me), 31.12 (N-Me), 29.81 (N-Me), 29.60 (N-Me), 29.84, 29.30, 29.14, 25.36, 24.99, 24.90, 24.66, 24.40, 23.81, 23.71, 23.40, 21.92, 21.83, 21.27, 20.37, 19.87 (15 Cs, 4 x $CH(CH_3)_2$ and 4 x $CH(CH_3)_2$, residues 4, 6, 9 and 10; 2 x $CH(CH_3)_2$, residues 5 and 11; CαH-CH_2-CH_3, residue 2), 18.72, 18.45 (2 x 2Cs, 4Cs, 2 x valine [$CH(CH_3)_2$]), 18.20, 16.90 (2Cs, 2 x CαH-CH_3, residues 7 and 8), 16.10 (CαH-CH[OH]-CH[side chain]-CH_3, residue 1), 9.93 (CαH-CH_2-CH_3, residue 2).

Formation of the Vinyl acid derivative (S3)

The methyl ester **S2** (260 mg, 0.196 mMol) was stirred in acetone (4 mL) and an aqueous solution of sodium hydroxide (2M, 2 mL). After 19 hours a white precipitate had formed and T.l.c. analysis (acetone : cyclohexane, 1: 1) showed the presence of one product (R_f 0.17) and some residual starting material/impurity (R_f 0.31). The acetone was removed from the reaction mixture and the aqueous layer left behind was washed with ethyl acetate. The aqueous layer was acidified with an aqueous solution of hydrochloric acid (1M) and washed again with ethyl acetate. The collected ethyl acetate layers were dried (magnesium sulfate), filtered and concentrated in vacuo to give a white/pale brown hygroscopic solid which was then diluted in acetonitrile and filtered again (eluant acetonitrile). The filtrate was finally concentrated in vacuo to give the acid derivative **3** (220 mg, 86%) as a white/pale brown hygroscopic solid.

HRMS (TOF MS ES^+): found 1330.8366 $[M+Na]^+$ $C_{68}H_{113}N_{11}O_{14}Na$ requires 1330.8173.

ν_{max} (thin film, KBr): 3418, 3315 (m, bumpy, CON-Hs, OH), 2961, 2936, 2873 (m, alkyl C-H), 1714 (m, conjugated C=OOH), 1627 (s broad, bumpy, C=Os, amide I), 1520 (m broad, bumpy, C=Os, amide II) cm^{-1}

NMR δ_H ($CDCl_3$, 500 MHz): (500 MHz, CDCl3) δ 3.51 (s, NMe, 3H), 3.37 (s, NMe, 3H), 3.23 (s, NMe, 3H), 3.09 (s, NMe, 3H), 3.08 (s, NMe, 3H), 2.68 (s, NMe, 3H), 2.65 (s, NMe, 3H).

NMR δ_C ($CDCl_3$, 125 MHz): 39.53, 34.19, 31.64, 31.35, 29.88 (2Cs), 29.61 (7 x N-Me).

Synthesis of Fmoc protected intermediate (S4)

HATU coupling reagent (230 mg, 0.6037 mMol) was added to a solution of the CsA acid **S3** derivative (395 mg, 0.3018 mMol), chloroform (10 mL) and triethylamine (168 µL) which had been stirring for 5 minutes under an atmosphere of nitrogen at room temperature. After a further 5 minutes 2-[2-(Fmoc-amino)ethoxy ethylamine hydrochloride (257 mg, 0.7083 mMol) was added to the stirring reaction mixture and left to react for 22.5 hours. LCMS analysis revealed the presence of the product in the reaction mixture. The reaction mixture was concentrated in vacuo and successively diluted in ethyl acetate and washed with an aq hydrochloric acid solution (1M). The collected organic layers were dried over magnesium sulphate, filtered and concentratedin vacuo to give a residue which was purified by flash column chromatography (chloroform to chloroform-methanol, 3%) to give the Fmoc derivative **S4** (406 mg, 83%) as a white hygroscopic solid.

HRMS (TOF MS ES^+): found 1638.9775 $[M+Na]^+$ $C_{87}H_{133}N_{13}O_{16}Na$ requires 1638.9891.

δ_H ($CDCl_3$, 500 MHz): 3.49 (s, NMe, 3H), 3.45 (s, NMe, 3H), 3.34 (s, NMe, 3H), 3.20 (s, NMe, 3H), 3.06 (s, NMe, 3H), 2.65 (s, NMe, 3H), 2.64 (s, NMe, 3H).

δ_C ($CDCl_3$, 125 MHz): 38.98, 33.97, 33.85, 31.77, 29.81 (2Cs), 29.66 (7 x N-Me).

Synthesis of the cyclosporin – PEG- amine derivative (S5).

To the FmoC protected CsA analogue **S4** (97 mg, 0.06 mMol) was added piperidine (0.5 mL), and the reaction was stirred overnight at rt. The piperidine was removed on a rotary evaporator and the residue purified by chromatography using 5-10% MeOH containing 2% 880 ammonia in CH_2Cl_2. This gave the intermediate amine **S5** (26 mg, 0.019 mMol, 31%) as yellow gum. This was used directly in the next step.

Synthesis of the fluorescein –PEG- CsA derivative (S6).

To the amine **S5** (22 mg, 18.6 mMol), 5/6-carboxyfluorescein (7 mg, 0.0187 mMol) and PyBOP (10 mg, 19 mMol) in CH_2Cl_2 (1 mL) was added diisopropylethylamine (9 mg, 13 µL, 76 mMol) and the reaction stirred overnight. The volatiles were removed on the rotary evaporator and the residue purified using reverse phase chromatography, C18, 5% MeOH to 95% MeOH in water. This gave the product **S6** (10 mg, 0.0057 mMol, 48%).

LCMS (ES+) 1775 ($M+Na^+$), 1752 ($M+H^+$). C18 >95%.

1-(10-Bromodecyl)quinolin-1-ium

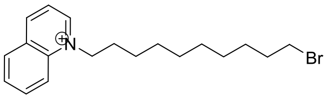

To a solution of quinoline (0.5 g, 3.87 mmol) in toluene was added 1,10-dibromodecane (1.74 g, 5.81 mmol) and this mixture was heated to 80°C for 48hrs. The mixture was allowed to cool before filtration. The solid was purified by column chromatography, eluting with 0-10% MeOH in DCM. The product was isolated as a clear oil (1.26 g, 93%).

^{1}H NMR (600 MHz, MeOD) δ 9.41 (dd, *J* = 5.8, 1.4 Hz, 1H), 9.21 (d, *J* = 8.3 Hz, 1H), 8.56 (d, *J* = 9.0 Hz, 1H), 8.44 (dd, *J* = 8.2, 1.4 Hz, 1H), 8.31 (ddd, *J* = 8.8, 7.0, 1.5 Hz, 1H), 8.15 – 8.01 (m, 2H), 5.09 (t, 2H), 3.43 (t, *J* = 6.7 Hz, 1H), 2.17 – 2.03 (m, 2H), 1.88 – 1.75 (m, 2H), 1.56 – 1.25 (m, 10H).

1-(10-((3-carboxy-4-(6-hydroxy-3-oxo-3H-xanthen-9-yl)benzoyl)oxy)decyl)quinolin-1-ium

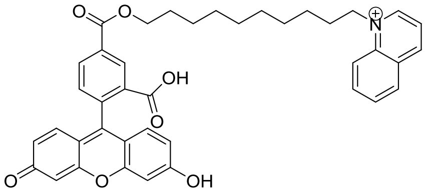

To a solution of 5-carboxyfluoroscein (0.065 g, 0.172 mmol) and sodium carbonate (0.04 g) in DMF was added dropwise a solution of 1-(10-bromodecyl)quinolinium (0.06 g, 0.172 mmol) in minimal DCM. This mixture was heated to 60°C overnight and was allowed to cool before dilution with DCM and washing with 1M citric acid. The organics were dried over $MgSO_4$ and concentrated under reduced pressure. The resultant orange solid was purified by column chromatography, eluting with 0-10% MeOH in DCM.

The product was isolated as a dark yellow solid (0.082 g, 75%).

^{1}H NMR (600 MHz, MeOD) δ 9.41 (d, *J* = 5.3 Hz, 1H), 9.16 (d, *J* = 8.3 Hz, 1H), 8.56 – 8.50 (m, 2H), 8.40 (d, *J* = 8.1 Hz, 1H), 8.35 (dd, *J* = 8.0, 1.3 Hz, 1H), 8.28 – 8.22 (m, 1H), 8.05 (dd, *J* = 8.3, 5.8 Hz, 1H), 8.01 (t, *J* = 7.6 Hz, 1H), 7.31 (d, *J* = 8.0 Hz, 1H), 6.67 (t, *J* = 3.5 Hz, 2H), 6.55 (d, *J* = 8.7 Hz, 2H), 6.53 – 6.48 (m, 2H), 5.13 – 5.00 (m, 2H), 4.38 (t, *J* = 6.4 Hz, 2H), 2.19 – 2.01 (m, 2H), 1.92 – 1.73 (m, 2H), 1.52 – 1.32 (m, 12H).
